# Characterizing and dissociating multiple time-varying modulatory computations influencing neuronal activity

**DOI:** 10.1371/journal.pcbi.1007275

**Published:** 2019-09-12

**Authors:** Kaiser Niknam, Amir Akbarian, Kelsey Clark, Yasin Zamani, Behrad Noudoost, Neda Nategh

**Affiliations:** 1 Department of Electrical and Computer Engineering, University of Utah, Salt Lake City, Utah, United States of America; 2 Department of Ophthalmology and Visual Sciences, University of Utah, Salt Lake City, Utah, United States of America; University of Pittsburgh, UNITED STATES

## Abstract

In many brain areas, sensory responses are heavily modulated by factors including attentional state, context, reward history, motor preparation, learned associations, and other cognitive variables. Modelling the effect of these modulatory factors on sensory responses has proven challenging, mostly due to the time-varying and nonlinear nature of the underlying computations. Here we present a computational model capable of capturing and dissociating multiple time-varying modulatory effects on neuronal responses on the order of milliseconds. The model’s performance is tested on extrastriate perisaccadic visual responses in nonhuman primates. Visual neurons respond to stimuli presented around the time of saccades differently than during fixation. These perisaccadic changes include sensitivity to the stimuli presented at locations outside the neuron’s receptive field, which suggests a contribution of multiple sources to perisaccadic response generation. Current computational approaches cannot quantitatively characterize the contribution of each modulatory source in response generation, mainly due to the very short timescale on which the saccade takes place. In this study, we use a high spatiotemporal resolution experimental paradigm along with a novel extension of the generalized linear model framework (GLM), termed the sparse-variable GLM, to allow for time-varying model parameters representing the temporal evolution of the system with a resolution on the order of milliseconds. We used this model framework to precisely map the temporal evolution of the spatiotemporal receptive field of visual neurons in the middle temporal area during the execution of a saccade. Moreover, an extended model based on a factorization of the sparse-variable GLM allowed us to disassociate and quantify the contribution of individual sources to the perisaccadic response. Our results show that our novel framework can precisely capture the changes in sensitivity of neurons around the time of saccades, and provide a general framework to quantitatively track the role of multiple modulatory sources over time.

## Author summary

The sensory responses of neurons in many brain areas, particularly those in higher prefrontal or parietal areas, are strongly influenced by factors including task rules, attentional state, context, reward history, motor preparation, learned associations, and other cognitive variables. These modulations often occur in combination, or on fast timescales which present a challenge for both experimental and modelling approaches aiming to describe the underlying mechanisms or computations. Here we present a computational model capable of capturing and dissociating multiple time-varying modulatory effects on spiking responses on the order of milliseconds. The model’s performance is evaluated by testing its ability to reproduce and dissociate multiple changes in visual sensitivity occurring in extrastriate visual cortex around the time of rapid eye movements. No previous model is capable of capturing these changes with as fine a resolution as that presented here. Our model both provides specific insight into the nature and time course of changes in visual sensitivity around the time of eye movements, and offers a general framework applicable to a wide variety of contexts in which sensory processing is modulated dynamically by multiple time-varying cognitive or behavioral factors, to understand the neuronal computations underpinning these modulations and make predictions about the underlying mechanisms.

## Introduction

In many brain areas, particularly ‘associative’ regions including parietal and prefrontal cortex, sensory processing is affected by various intrinsic or extrinsic nonsensory covariates such as task or context variables, attention, learned associations, motor preparation, or cognition-related control signals. The fact that multiple such variables may simultaneously modulate sensory activity, and that their influence can change rapidly over the course of a task, poses challenges for precise experimental or computational quantification of their relative contributions to neuronal responses. In this paper, we develop a data-driven computational framework, which provides a rich statistical description of encoding time-varying sensory information by capturing and dissociating multiple time-varying modulations on the order of milliseconds.

We develop and test our model in the context of changes in visual sensitivity around the time of eye movements, which is an exemplar of such time-varying modulatory computations. There is a considerable literature demonstrating that visual neurons’ responses are modulated during rapid eye movements, known as saccades; these neurophysiological changes presumably underlie the biases in perception which also occur around the time of eye movements. Even during fixation, extrastriate cortical responses can show complex spatiotemporal dynamics which encode information about the stimulus [1]. Various types of perisaccadic response modulations have been reported. For example, many studies have shown that visual neurons lose their sensitivity to stimuli appearing in their receptive fields (RFs) shortly before a saccade (saccadic suppression) [2–7]. Other studies have demonstrated that visual neurons may preemptively shift their RF to the post-saccadic RF (future field remapping, or FF-remapping) [8–15], or to the saccade target (saccade target remapping, or ST-remapping) [16–18], even before a saccade is initiated. Moreover, there are several reports suggesting that the spatial distribution of the population of visual neurons’ RFs changes during or just prior to a saccade [19–21]. Taken together, these findings indicate that the perisaccadic responses evoked in visual neurons are modulated by several sources, i.e. stimuli perisaccadically presented at multiple locations in the visual field contribute to driving neurons’ responses.

Although existing experimental data identify those sources contributing to perisaccadic response modulation, they do not quantitatively characterize the contribution of each modulatory source to the response, alone or in combination with the other sources. A full understanding of visual perception during saccades may require the ability to reconstruct the visual scene across an eye movement based on neural activity, and this in turn necessitates a comprehensive understanding of how each modulatory source individually contributes to the perisaccadic representation, how the contributions of multiple sources are combined, and more importantly, how knocking out one of the sources may impact the reconstruction of the scene based on neural activity. Some of the limitations of the experimental data are practical: the short timescale on which the perisaccadic changes take place, combined with the limited number of trials that can be recorded in a single recording session, do not permit a full test of the contribution of different sources at each time point and in all combinations. This is where computational models come into play with two important roles, (1) making predictions of the neural responses to a wide variety of stimuli, and, (2) providing a quantitative description of how the modulatory sources contribute to response generation at different times relative to a saccade.

The fast changes in sensitivity around the time of saccades pose challenges for computational as well as experimental approaches. These changes make the stimulus-response relationship time-variant and create nonstationary responses and computations. This property makes many existing computational approaches, which are often based on time-invariant assumptions about the neural system, not applicable for modeling the nonstationary responses observed during a saccade. The approaches that have commonly been used to characterize nonstationary responses can be divided into three main categories. In the first approach, separate models are applied to several (overlapping or nonoverlapping) time intervals assuming that the stimulus-response relationship remains constant within each of those intervals [22–26]. This approach is more suitable for providing coarse snapshots of the neural states rather than analyzing how the states evolve over time. To address this limitation, the second approach provides methods for estimating the temporal variations of the model parameters to keep track of the temporal evolution of the underlying system. Among the methods using this approach, adaptive filtering solutions have widely been used in neural data analysis, especially in studying the temporal evolution of the spatiotemporal and spectrotemporal receptive field of neurons [27–32]; however, depending on the size of their parameter space, these models require a large amount of data for their parameter estimation, and as a result they fall short in the cases where the evolution of the underlying system happens on a very short timescale, which is the case in perisaccadic studies. The third approach uses state-space methods such as linear dynamical systems [33–35] or hidden Markov models [36–41], in which the next state of the system is determined based on its current state and its input. Although these methods have been successful in modeling dynamic neural data and especially for neural decoding applications, similar to adaptive filters, they require a large amount of data to work, which makes them insufficient for the resolution or precision required for modeling the perisaccadic responses. To address the need for a quantitative means to study nonstationary responses across a saccade, we recently developed an extension of the widely-used generalized linear models (GLMs) [42–47], termed the nonstationary generalized linear model (NSGLM, referred to as the N-model in this article) to describe response dynamics in visual neurons across a saccade [48]. The multiplicative spatiotemporal gain kernels introduced in the N-model recovered the rapid eye displacement signal and the resulting nonstationarity in responses solely based on the statistical relationship between the stimulus and response across a saccade. However, the N-model structure remained limited to the types of nonstationarity that could be described by gain factors modulating the filtered input stimuli, and could not be generalized to explain the range of response nonstationarities and their underlying modulatory computations observed during saccades.

None of the existing models are capable of tracking the dynamic changes in sensitivity which accompany saccades on millisecond timescales. In this paper, we present a new nonstationary approach to develop a sparse-variable generalized linear model (referred to as the S-model), which is capable of tracking the rapid changes occurring in the stimulus-response relationship across a saccade in the middle temporal (MT) cortex of nonhuman primates. The S-model is composed of a set of time-varying stimulus kernels which represent the time-varying spatiotemporal sensitivity of a neuron. Building on the success of the S-model in predicting perisaccadic responses, a circuit-inspired factorized version of the S-model (referred to as the F-model) was developed in order to dissociate and to emphasize the role of individual sources contributing to perisaccadic response modulation. In the F-model, each stimulus kernel is decomposed into a parsimonious set of multiple modulatory sources, combined to describe the spatiotemporal receptive field of the neuron. The F-model not only accurately captures the perisaccadic changes in neural sensitivity, but also provides a tractable computational model by which the contribution of various modulatory sources can be dissociated. This temporally precise, quantitative decomposition of a neuron’s perisaccadic responses offers unique opportunities for quantifying perisaccadic modulations and testing the perceptual effects of various perisaccadic modulatory sources. Furthermore, the computational framework can be applied to quantify and dissociate the effects of multiple modulatory factors on neuronal responses in a variety of brain areas and behavioral tasks.

## Results

### Time-varying modulation of neuronal responses: Saccades modulate the responses of MT neurons

**Fig 1:**
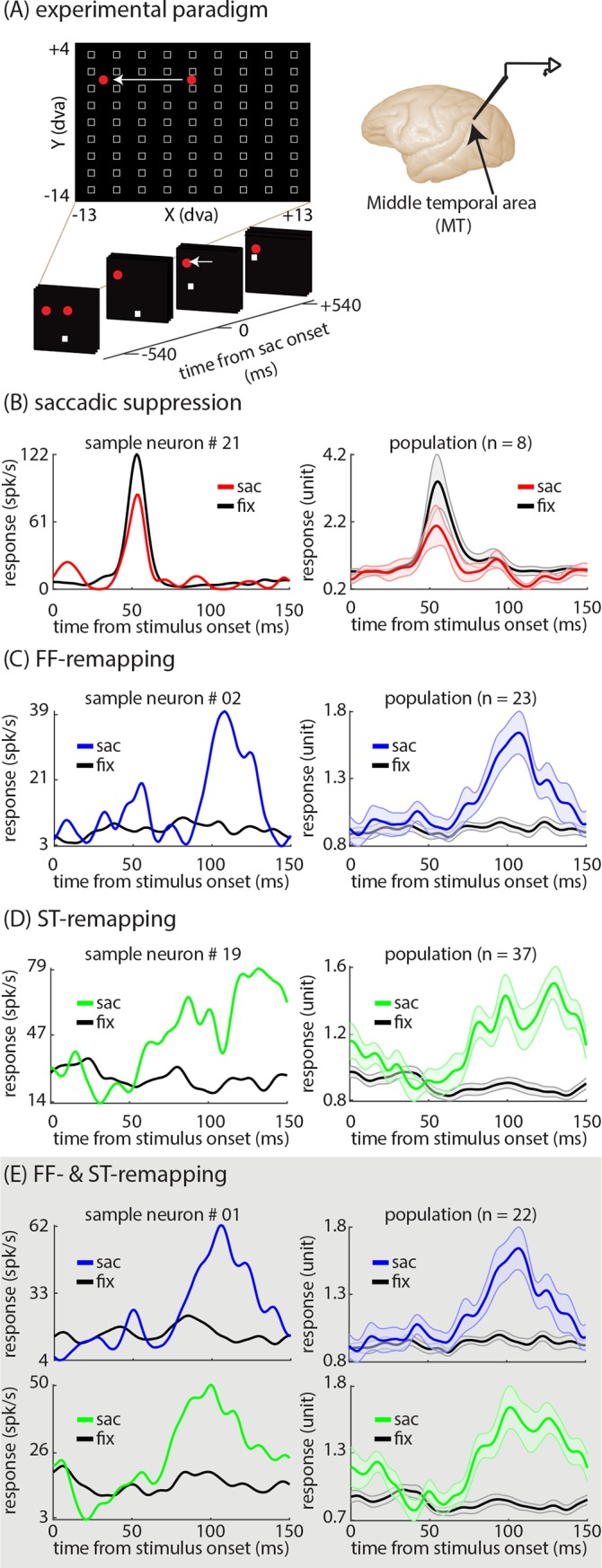
Saccades alter the visual sensitivity of MT neurons. **(A) Illustration of the visually-guided saccade task with visual probes**. Monkeys fixated on a central fixation point (FP: central red dot), and a target point (ST: peripheral red dot) appeared 10 degrees away horizontally. Then, after a randomized time interval, the fixation point disappeared, cueing the monkeys to saccade to the target. Throughout the task, a series of randomly located probes were presented on the screen in a 9 by 9 grid of possible locations (white squares) such that at each time-point there was only one probe on the screen. The x-axis and y-axis show the extent of the probe grid in dva when the fixation point and saccade target are located at (0,0) and (-10,0) dva, respectively. Along the axis on the bottom, a sequence of sample snapshots of the visual scene projected on the screen is shown as a function of time to saccade for the duration of an example trial (white arrow indicates eye movement). The activity of MT neurons was recorded using linear array electrodes. **(B) Saccadic suppression in an example neuron and the modulated subpopulation**. The left panel shows the response of a sample MT neuron to a stimulus presented inside its RF when the stimulus onset is shortly before a saccade (red, perisaccadic: -30 to 0 ms from saccade) compared to when it is long before a saccade (black, fixation: -500 to -100 ms from saccade). The right panel represents the average of the normalized perisaccadic (red) and fixation (black) responses of 8 MT neurons with significant saccadic suppression. The shaded area indicates the mean ± SE. **(C) FF-remapping in an example neuron and the modulated subpopulation**. The left panel shows the response of a sample MT neuron to a stimulus presented inside its FF when the stimulus onset is shortly before a saccade (blue, perisaccadic: -50 to 0 ms from saccade) compared to when it is long before a saccade (black, fixation). The right panel represents the average of the normalized perisaccadic (blue) and fixation (black) responses of 23 MT neurons with significant FF-remapping. **(D) ST-remapping in an example neuron and the modulated subpopulation**. The left panel shows the response of a sample MT neuron to a stimulus presented around the ST when the stimulus onset is shortly before a saccade (green, perisaccadic: -50 to 0 ms from saccade) compared to when it is long before a saccade (black, fixation). The right panel represents the average of the normalized perisaccadic (green) and fixation (black) responses of 37 MT neurons with significant ST-remapping. **(E) FF- and ST-remapping in an example neuron and the modulated subpopulation**. The left panels show the response of a sample MT neuron to a stimulus presented inside its FF (top) and around the ST (bottom) when the stimulus onset is shortly before a saccade (blue and green, perisaccadic: -50 to 0 ms from saccade) compared to when it is long before a saccade (black, fixation). The right panels represent the average of the normalized perisaccadic (blue and green) and fixation (black) responses of 22 MT neurons with significant FF- and ST-remapping. The enclosing grey box indicates the co-existence of both remapping effects in individual neurons.

The principal objective for the new computational framework developed in this study is to provide a statistical framework that will capture the encoding of time-varying information in higher brain areas. The desire for such a model was motivated by our findings about the perisaccadic response properties of neurons in area MT of macaque monkeys. The activity of 41 single neurons in MT cortex was recorded while animals performed a visually-guided saccade task with probe stimuli (Fig 1A). Each stimulus appeared on the screen for only 7 milliseconds (ms), allowing a high spatiotemporal resolution mapping of the neurons’ visual sensitivity. Neurons exhibited several types of changes in perisaccadic sensitivity, including saccadic suppression, FF-remapping, and ST-remapping. Fig 1B shows the saccadic suppression effect in an example neuron and the subpopulation of significantly modulated neurons (n = 8). Perisaccadic visual responses to a stimulus in the original RF are reduced compared to responses during fixation. For the example neuron, the average response to an RF stimulus dropped from 60.89 ± 2.82 (mean ± SE) spk/s during fixation to 40.49 ± 12.64 (mean ± SE) spk/s when the stimulus appeared just prior to saccade onset (example neuron, p < 0.001). For the subpopulation of neurons with significant saccadic suppression (see Methods), the average normalized response dropped from 2.53 ± 0.37 to 1.51 ± 0.29 (mean ± SE). Fig 1C shows the FF-remapping effect in an example neuron and the subpopulation of significantly modulated neurons (n = 23). During fixation there is no response to stimuli appearing in the FF; however, during the perisaccadic period neurons respond to stimuli in the FF. Note that the perisaccadic FF response occurs at longer latency than RF responses (neurons responding 50–75 ms after stimulus onset for the RF, vs. 80–150 ms after stimulus onset for the FF). For the example neuron, the average late response to an FF stimulus increased from 7.90 ± 1.02 (mean ± SE) spk/s during fixation to 18.42 ± 3.56 (mean ± SE) spk/s perisaccadically (example neuron, p < 0.001). For the subpopulation of neurons with significant FF-remapping, the average normalized response to FF stimuli increased from 0.90 ± 0.03 to 1.30 ± 0.08 (mean ± SE). Fig 1D shows the ST-remapping effect in an example neuron and the subpopulation of significantly modulated neurons (n = 37). During fixation there is no response to stimuli appearing around the ST; however, during the perisaccadic period neurons respond to stimuli around the ST. Like the FF-remapping effect, the perisaccadic ST response occurs at longer latency than RF responses. For the example neuron, the average late response to a stimulus near the ST increased from 23.80 ± 1.77 (mean ± SE) spk/s during fixation to 64.64 ± 7.91 (mean ± SE) spk/s perisaccadically (example neuron, p < 0.001). For the subpopulation of neurons with significant ST-remapping, the average normalized ST response increased from 0.83 ± 0.03 to 1.35 ± 0.06 (mean ± SE). The RF, FF, and ST effects could occur in different combinations or relative strengths across neurons. Fig 1E shows the FF- and ST-remapping effects for an example neuron and the subpopulation of neurons exhibiting both effects (n = 22). For the example neuron, the average late response to a stimulus in the FF increased from 15.83 ± 0.91 (mean ± SE) spk/s during fixation to 39.10 ± 5.14 (mean ± SE) spk/s perisaccadically (example neuron, p < 0.001), and the average response to a stimulus near the ST increased from 16.15 ± 0.94 (mean ± SE) spk/s during fixation to 36.17 ± 4.49 (mean ± SE) spk/s perisaccadically (example neuron, p < 0.001). For the subpopulation of neurons with significant FF- and ST-remapping, the average normalized FF response increased from 0.90 ± 0.03 to 1.32 ± 0.08 (mean ± SE), and the average normalized ST response increased from 0.80 ± 0.03 to 1.46 ± 0.08 (mean ± SE).

### Time-varying encoding models: The new model framework describes the time-varying perisaccadic responses of MT neurons

A set of novel variants on a classical GLM framework were developed with the goal of capturing perisaccadic modulations in visual sensitivity. The principal idea for using a GLM framework as a base structure in this study has several folds: (i) providing a rich, statistical description of stimulus-response relationship, (ii) computational tractability of the estimation procedure, (iii) providing biologically plausible response generation and modulation components, and finally, (iv) its flexibility to incorporate a variety of external and internal covariates depending on the experimental design and the estimation or prediction tasks at hand, which is crucial in this study. The structure of the models is largely similar (see Methods), with the exception of the stimulus kernels which are used to represent changes in spatiotemporal sensitivity. In the sparse-variable generalized linear model (S-model), the stimulus-response relationship in a neuron is characterized by time-varying stimulus kernels representing the time-dependent spatiotemporal receptive field profile of the neuron. The kernels of the S-model were estimated by maximizing the likelihood of the observed spikes under the instantaneous firing rate predicted by the model over the training set, and validating over the validation set. We next produced a parsimoniously factorized version of the S-model (F-model), in which the fitted kernels of the S-model were represented as a mixture of three time- and delay-dependent spatial skewed Gaussian kernels, where each captures the modulation arising from one of the RF, FF, or ST sources (corresponding to the modulations observed in perisaccadic responses as detailed in the last subsection). Finally, an aggregate model (A-model) was constructed by fitting the S-model components ten times (over subsets each including a randomly selected 65% of the data), creating an F-model from each of these S-models, and then taking the average of the F-model components, in order to obtain a model with kernels reflecting the entire dataset.

**Fig 2:**
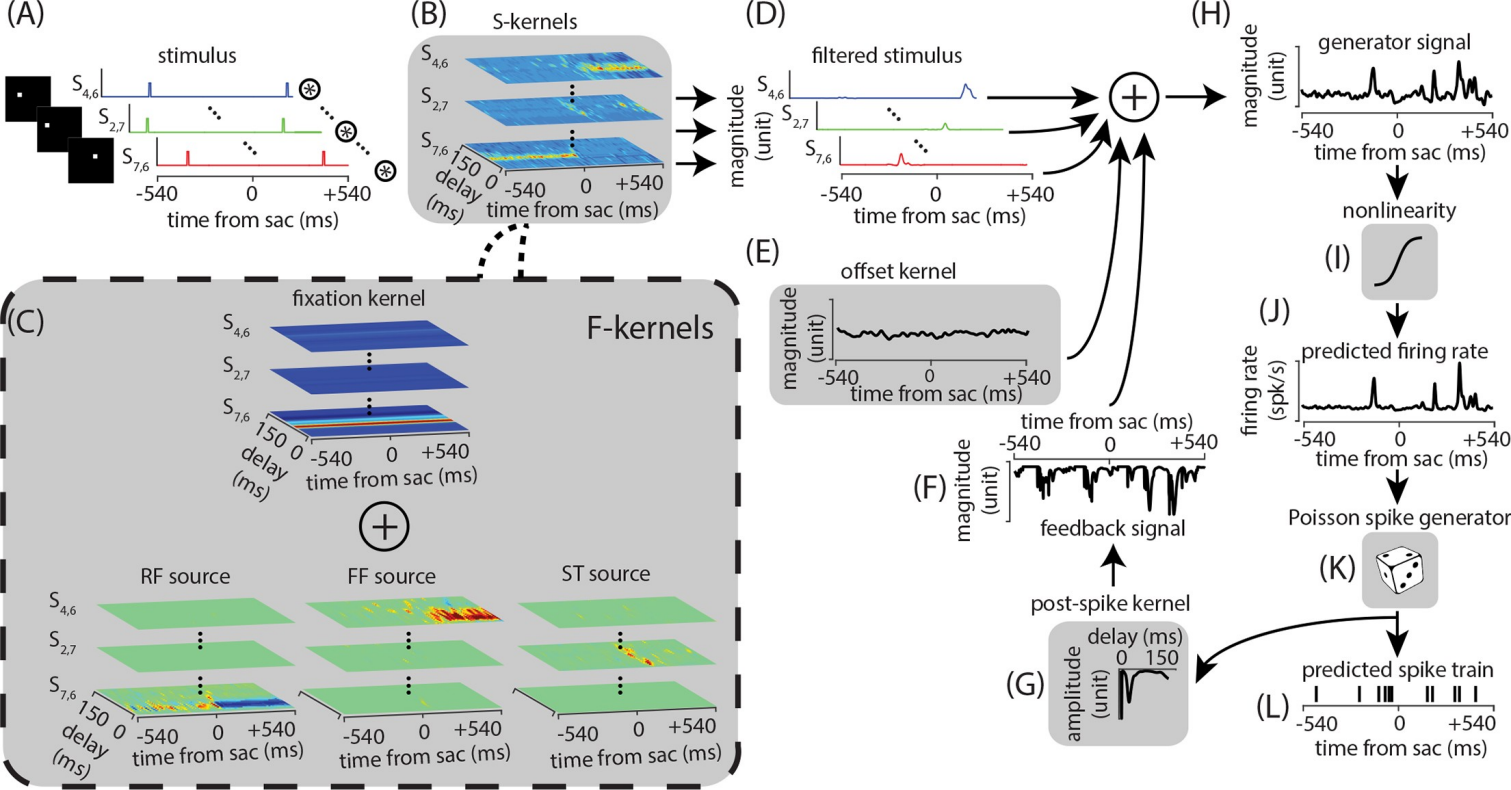
Schematic of the S- and F-model structure and fitted components. Both models get the visual stimulus as input, and generate a prediction for the firing rate of the neuron. The input to the model is a set of temporal sequences of the stimulus along each spatial dimension, shown in (A) for three example locations (S_4,6_, S_2,7_, and S_7,6_). These inputs are convolved with time-varying kernels representing the time-varying spatiotemporal receptive field of the neurons. In the S-model, each probe location has its own time-varying kernel (S-kernels in (B)). In the F-model, a kernel associated with each probe location is approximated by a combination of the RF, FF, and ST modulatory sources added to a fixation kernel (F-kernels in (C)). The outputs of the convolution of the inputs and S-/F-kernels (D) are then summed with an offset kernel (E) as well as a feedback signal (F) via the post-spike kernel (G), and the resulting generator signal (H) is passed through a sigmoidal nonlinear function (I) to predict the neuron’s instantaneous firing rate (J), which will be used to generate the neuron’s spiking activity (L) via a conditionally Poisson process (K). An example spike train as generated by the model is illustrated here.

Fig 2 shows the full structure of the S- and F-models. Both models specify the probabilistic relationship between a sequence of input stimuli, defined in time and space, and the measured neural spike trains on the scale of single trials. Inputs to the models (Fig 2A) are convolved with a set of time-varying stimulus kernels (Fig 2B and 2C). For the S-model, each probe location has its own time-varying kernel (S-kernels, Fig 2B). In the F-model, each S-kernel is optimally approximated by a combination of the RF, FF, and ST modulatory sources added to a fixation kernel (F-kernels, Fig 2C). The output (Fig 2D) is then summed with an offset kernel (Fig 2E) and the feedback signal (Fig 2F) generated by the post-spike kernel (Fig 2G). The resulting generator signal (Fig 2H) is then passed through a nonlinearity (Fig 2I) to generate the instantaneous firing rate (Fig 2J). A Poisson spike generator (Fig 2K) is used to generate the spiking response (Fig 2L).

### The time-varying encoding models accurately predict single trial spike response modulations across saccades

**Fig 3:**
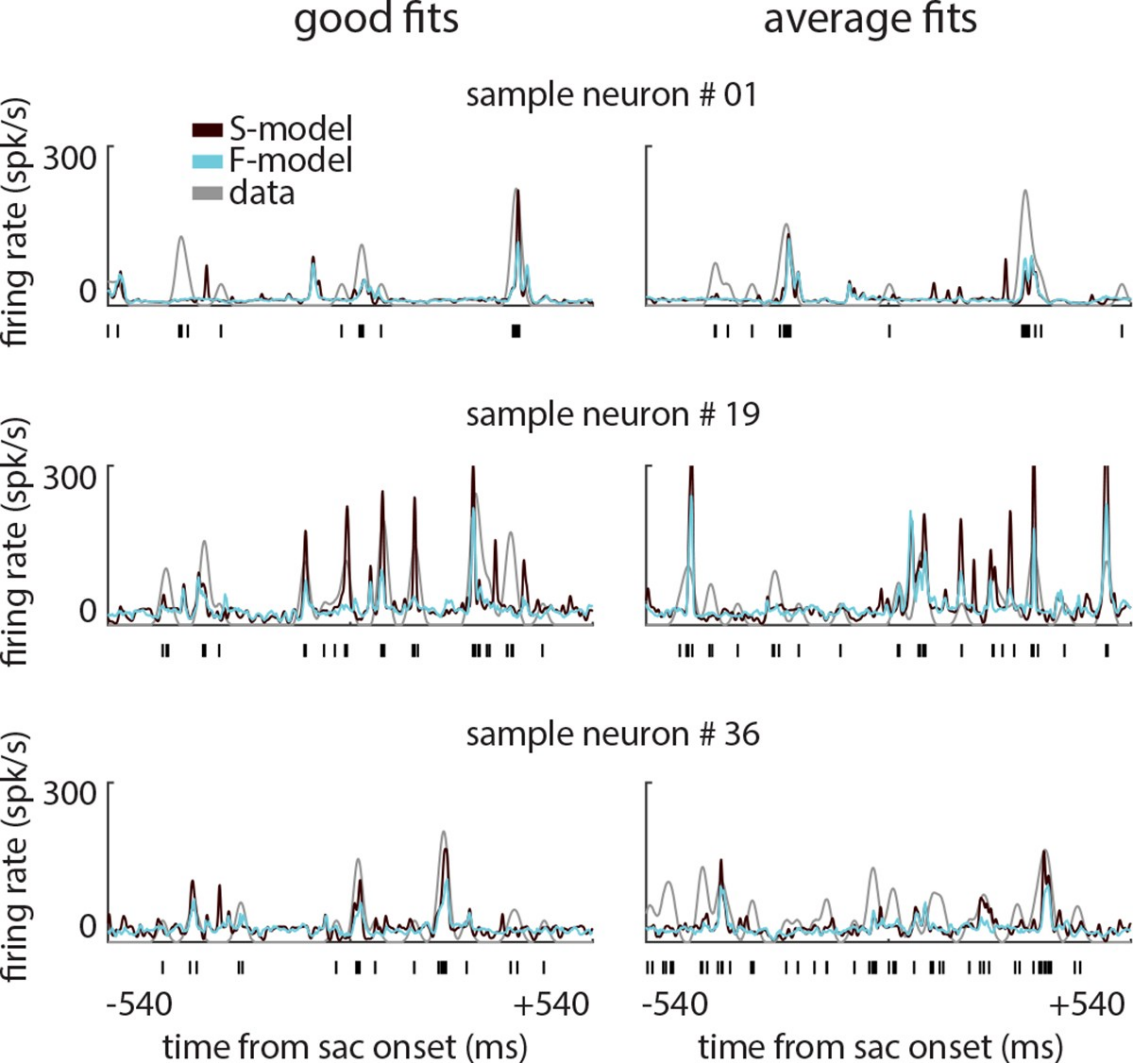
Single trial firing rate prediction using the S- and F-models. Results are shown for 3 sample MT neurons. Each row shows two example trials for a single neuron. Black vertical bars at the bottom of x-axis indicate the experimentally measured spike trains. Grey traces show the estimated firing rate obtained by smoothing the recorded spike trains with a Gaussian with full width at half-max (FWHM) of 33 ms, and brown and cyan traces show the model-predicted firing rate for that trial using the S-, and F-models, respectively. Left column shows example trials for which the prediction accuracy was highest, and right column shows example trials with median prediction accuracy.

Fig 3 illustrates the ability of the S- and F-models to reproduce the time course of the neural activity across trials. Two sample trials are shown for three neurons indicating how well the models captured the instantaneous firing rate of the neurons on the unseen data. The trials on the left show examples of high prediction accuracy (from top to bottom, Δ*LL*/*spk* = 1.12, 0.80, and 0.63 bits/spk for the S-model; and Δ*LL*/*spk* = 0.81, 0.47, and 0.55 bits/spk for the F-model), and the trials on the right show examples of median prediction accuracy (from top to bottom, Δ*LL*/*spk* = 0.46, 0.41, and 0.19 bits/spk for the S-model; and Δ*LL*/*spk* = 0.41, 0.34, and 0.05 bits/spk for the F-model) for each neuron and model. (The A-model was omitted from this analysis as it uses all data when aggregating over the multiple F-model fits, and so, there is no unseen data for the A-model.)

**Fig 4:**
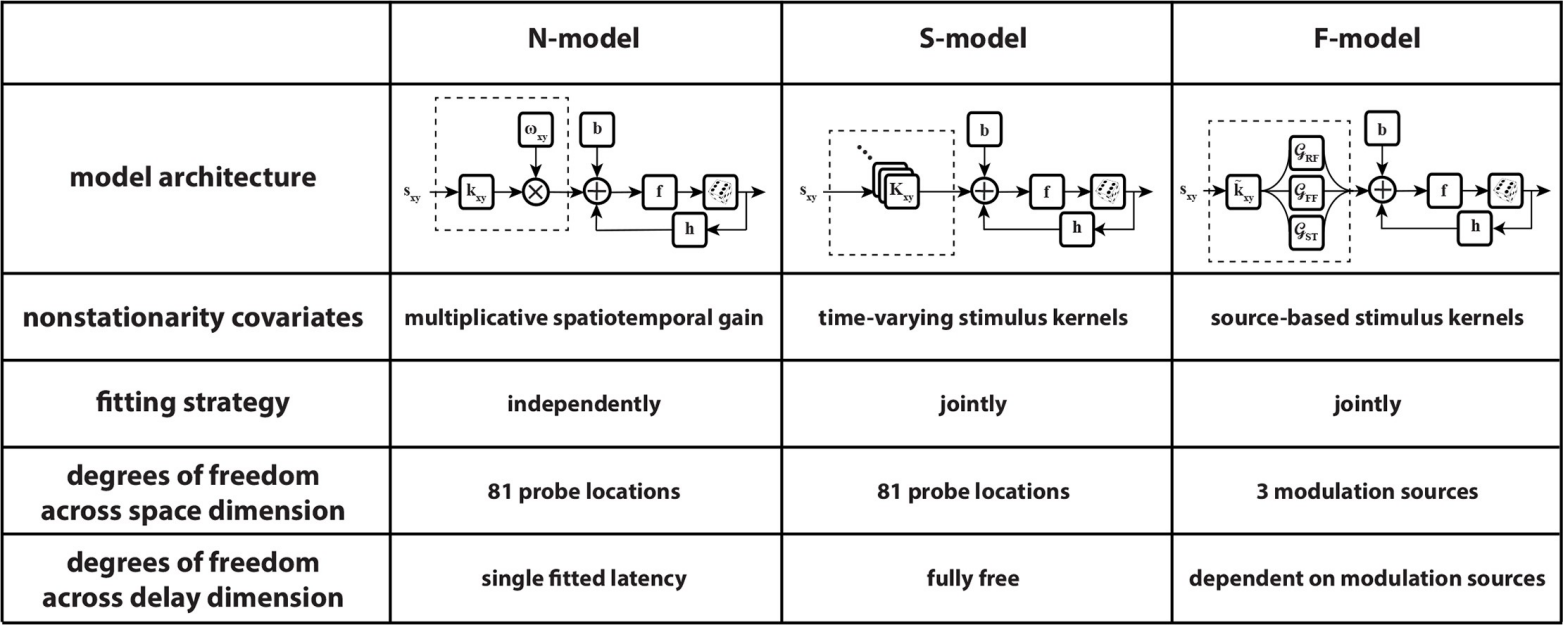
Overview of models’ architecture and properties. The table illustrates the models’ architecture and properties for the N-, S-, and F-models.

A summary of the models’ structures and key properties is presented in Fig 4. The N-model was described in a previous publication [48] and is presented here for the purpose of comparison. The N-, S-, and F-models are all variations on the well-known GLM structure, using different approaches to capture the nonstationarities existing in the perisaccadic responses. In terms of model components (Fig 4, first row), the N-model has a gain kernel and time-invariant stimulus kernel at each probe location, as well as post-spike and offset kernels, while the S-model has time-variant stimulus kernels at each probe location, a post-spike kernel, and an offset kernel. The components of the F-model were three skewed Gaussian kernels, and post-spike and offset kernels taken from the S-model. In the N-model, the nonstationarity is modeled by multiplying a space- and time-varying gain by the result of the convolution of input stimuli and time-invariant stimulus kernels (Fig 4, second row). Although successful in capturing the shift of spatial sensitivity following an eye movement, the N-model fails to describe the perisaccadic responses arising from a range of modulatory computations beyond an instantaneous gain mechanism, e.g. changes in response latency. The S-model deals with this issue by employing time-varying stimulus kernels, providing more degrees of freedom for the model; in effect, the S-model can be considered as a set of GLMs, each one corresponding to a single time point and all fitted simultaneously. The S-model accurately characterizes the observed perisaccadic modulations, but does not lend itself easily to a mechanistic level interpretation. To find a way to interpret the S-kernels, and identify and dissociate possible sources that give rise to the spatiotemporal sensitivities revealed by the S-kernels, the fitted stimulus kernels of the S-model were factorized into sources of modulation in the F-model, such that stimulus kernels were reconstructed by a combination of three modulatory sources added to a fixation kernel representing the neuron’s behavior during fixation. This allowed the interpretation of changes in responses (especially perisaccadic responses) as resulting from changes in the characteristics of a few modulatory sources. Due to the structure of the N-model, the model components responsible for response generation (stimulus kernels) and response modulation (gain kernels) are fitted independently, while these components are interwoven in the S- (two-dimensional stimulus kernels) and F-models (skewed Gaussian kernels) (Fig 4, third row). While in the N- and S-models there are separate stimulus kernels corresponding to separate probe locations, the modulatory sources in the F-model have a spatial profile, and so, the spatiotemporal receptive field structure of the neuron in the F-model is described by just three sources at each time point relative to the saccade instead of a set of stimulus kernels (Fig 4, fourth row). Moreover, while the response latency cannot vary across time in the N-model, and can vary at each time and probe location in the S-model, the F-model determines the response latencies based on three modulatory sources (Fig 4, fifth row).

**Fig 5:**
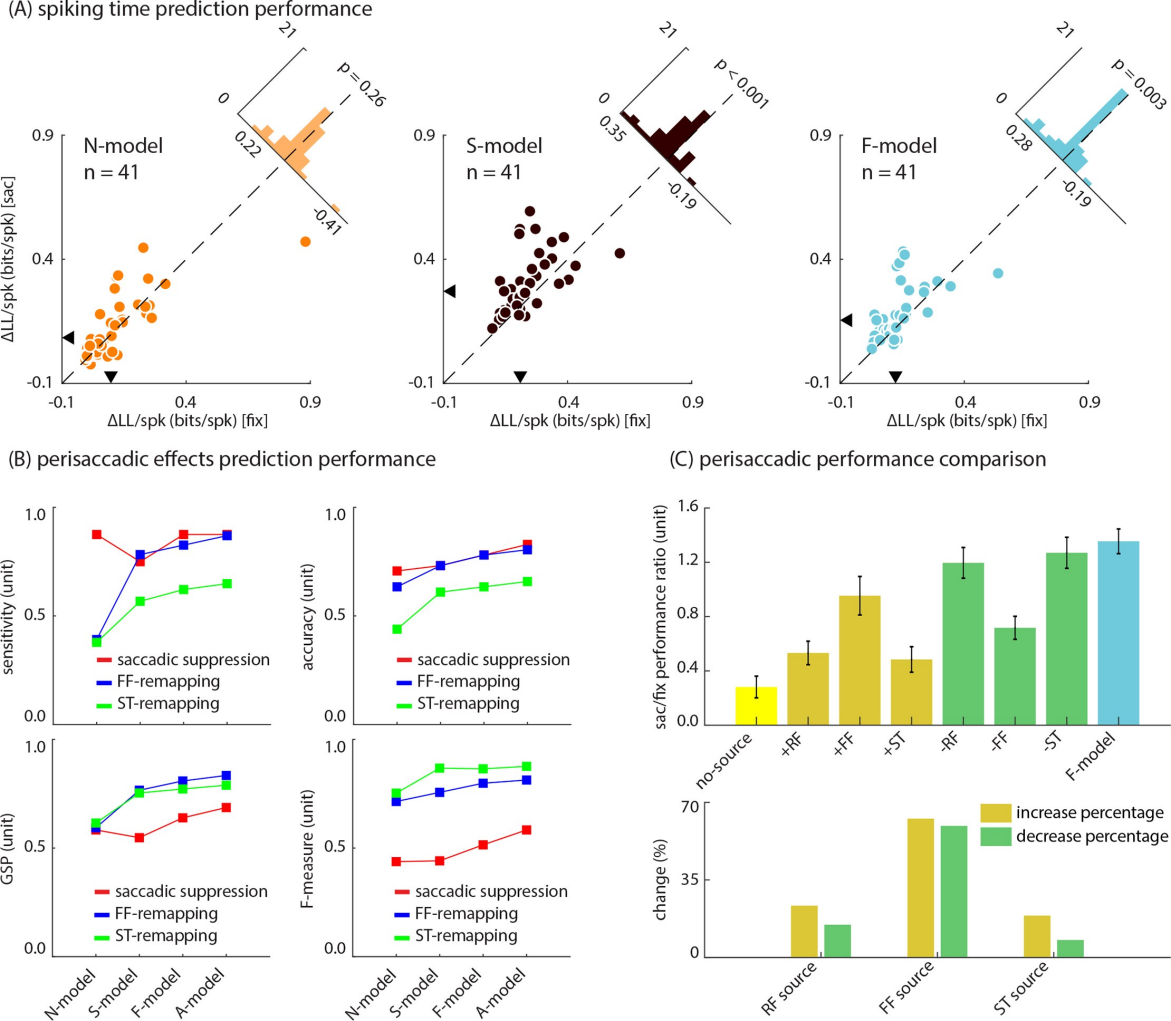
Performance comparison across models. **(A) Comparison of each models’ performance between fixation and perisaccadic periods**. Each scatterplot shows one model’s performance, measured in terms of Δ*LL*/*spk*, for 41 neurons during the fixation and perisaccadic period. Histograms in the upper right show the population distribution of change in performance between the fixation and perisaccadic period. Arrowheads along the x- and y-axes mark the medians of each distribution. **(B) S-, F-, and A-models capture the observed perisaccadic changes in neural responses better than the N-model**. Plots compare the ability of N-, S-, F-, and A-models to capture each neuron’s perisaccadic response properties, evaluated over the population of 41 neurons. Plots show 4 different measures of classification performance (sensitivity, accuracy, the geometric mean of sensitivity and precision, and F-measure), for each of three perisaccadic effects (saccadic suppression, red; FF-remapping, blue; and ST-remapping, green). **(C) Contributions of FF, ST, and RF modulations to perisaccadic performance**. Plot on the top compares the perisaccadic to fixation performance ratio of the F-model with different variants of the F-model. The error bars show the standard errors. Plot on the bottom summarizes these results in terms of percentage changes (relative to maximum change, F-model–no-source model) after adding or eliminating one of the sources.

The ability of the N-, S-, and F-models to predict single spike trains (over test data) during the fixation vs. perisaccadic period is compared in Fig 5A (again the A-model was omitted from this analysis for the same reason described in Fig 3). The N-model is no better at predicting the spike times during the perisaccadic period compared to the fixation period (Δ*LL*/*spk* = 0.12 ± 0.02 (mean ± SE) bits/spk during fixation vs. 0.13 ± 0.02 (mean ± SE) bits/spk during saccade, p-value = 0.26). For the S-model, the prediction accuracy increases in the perisaccadic period (Δ*LL*/*spk* = 0.24 ± 0.02 (mean ± SE) bits/spk during fixation vs. 0.30 ± 0.02 (mean ± SE) bits/spk during saccade, p-value < 0.001). The F-model, despite being based on an approximation of the S-model, also shows greater prediction accuracy during the perisaccadic period compared to fixation (Δ*LL*/*spk* = 0.14 ± 0.01 (mean ± SE) bits/spk during fixation vs. 0.18 ± 0.02 (mean ± SE) bits/spk during saccade, p-value = 0.003). These improvements reflect the efficiency of the fitting strategy tailored to capture dynamic aspects of the response. The richer architectures of the S- and F- models allowed them to capture dynamic changes in the neuron’s spatiotemporal response as a function of time to the saccade, and as a result, both better predict the responses compared to the N-model. Both the S-, and F-models outperform the N-model during both the fixation (S-model vs. N-model: p-value < 0.001; F-model vs. N-model: p-value = 0.001) and perisaccadic (S-model vs. N-model: p-value < 0.001; F-model vs. N-model: p-value < 0.001) periods. In all comparisons, the statistical significance was determined by the Wilcoxon signed-rank test (n = 41 neurons).

As seen in Fig 5A, the F-model trails the S-model in terms of spiking time prediction, which is not surprising given the greater number of variables in the S-model; however, when it comes to the previously reported perisaccadic modulations (i.e., saccadic suppression, FF-remapping, and ST-remapping), the F-model performs as well as the S-model. In order to demonstrate this point, Fig 5B examines the ability of all four models to reproduce the saccadic suppression, FF-remapping, and ST-remapping effects (over all recorded trials, not only test trials, to avoid a bias toward a subset of data). For this analysis, neurons were categorized as displaying or not displaying each of three perisaccadic effects using their recorded responses. Then, the models’ predictions were used to re-classify neurons into the same categories, and the response-based and model-based classifications were compared using four different measures of classification performance (sensitivity, accuracy, the geometric mean of sensitivity and precision, and F-measure). The predictions of each model were compared using the one-tailed mid P-value McNemar test [49], with the null hypotheses that the S-/F-/A- and N-models have equal performance in terms of reproducing each of three perisaccadic effects (P-values less than 0.05 were considered a rejection of the null hypothesis). For the FF-remapping effect, the A-model significantly outperforms the N-model (p = 0.048); the S- and F- models did not significantly outperform the N-model (S-model vs. N-model: p = 0.17, and F-model vs. N-model: p = 0.08). The S-, F-, and A-models all significantly outperform the N-model in terms of reproducing the ST-remapping effect (S-model vs. N-model: p = 0.004, F-model vs. N-model: p = 0.006, and F-model vs. N-model: p = 0.006). Finally, we did not find any significant difference between the S-, F-, and A-models and N-model in terms of reproducing the saccadic suppression effect (S-model vs. N-model: p = 0.38, F-model vs. N-model: p = 0.17, and F-model vs. N-model: p = 0.11), indicating that a global change in gain is sufficient to reproduce the saccadic suppression effect.

Additionally, in order to compare the ability of the S- and F-models to accurately predict responses specifically during the perisaccadic period, we calculated the ratio of perisaccadic performance to fixation performance (in terms of Δ*LL*/*spk* over test data) for the S- and F-models. Since adding additional variables is generally expected to improve model performance, during both the fixation and perisaccadic periods, this ratio of perisaccadic to fixation performance was used to quantify the added perisaccadic predictive value of the model regardless of overall changes due to the degrees of freedom. The S-model has a lower perisaccadic to fixation ratio compared to the F-model (1.33 ± 0.99 (median ± SE) for the S-model vs. 1.51 ± 0.25 (median ± SE) for the F-model, Wilcoxon signed‐rank p‐value = 0.003), indicating that although overall performance is higher due to the S-model’s greater degrees of freedom, the F-model does better at specifically reproducing perisaccadic changes in the response. In order to assess the contribution of individual sources, represented by time- and delay-dependent spatial skewed Gaussian kernels, to the F-model’s performance, different variants of the F-model were created (as detailed in the Methods section). These variants included models in which only one of the three sources was included (+RF, +FF, and +ST), models in which one of the sources was eliminated (-RF, -FF, or -ST), and one in which all three modulations were eliminated (no-source). A linear regression was conducted in which the slope of a line fitted to the perisaccadic vs. fixation performance for the population of 41 neurons was considered as an estimate for the perisaccadic to fixation ratio of each model. In order to evaluate the role of each source in the model’s perisaccadic performance, the ratios of perisaccadic to fixation performance were compared across the model variants described above. As seen in Fig 5C, top panel, the no-source model has the lowest perisaccadic to fixation performance ratio (0.28 ± 0.08). The perisaccadic to fixation performance ratio increases when any individual source is added to the model (+RF: 0.53 ± 0.09, +FF: 0.95 ± 0.14, +ST: 0.48 ± 0.09). The F-model which incorporates all three sources has the highest perisaccadic to fixation performance ratio (1.35 ± 0.09), and eliminating any individual source decreases the perisaccadic to fixation performance ratio (-RF: 1.20 ± 0.11, -FF: 0.72 ± 0.08, -ST: 1.27 ± 0.11). Fig 5C, bottom panel, summarizes these results in terms of what percent of the total improvement (the difference between the F-model and the no-source model) is present after adding or eliminating each of the sources. As seen, adding the RF, FF, or ST source improves the perisaccadic to fixation ratio by 23.38%, 62.65%, and 18.89%, respectively, and eliminating the RF, FF, or ST source decreases the ratio by 14.79%, 59.38%, and 7.87%, respectively.

From now on, “model” refers to the A-model whenever not otherwise specified.

### The model captures the spatial and temporal characteristics of the perisaccadic effects

**Fig 6:**
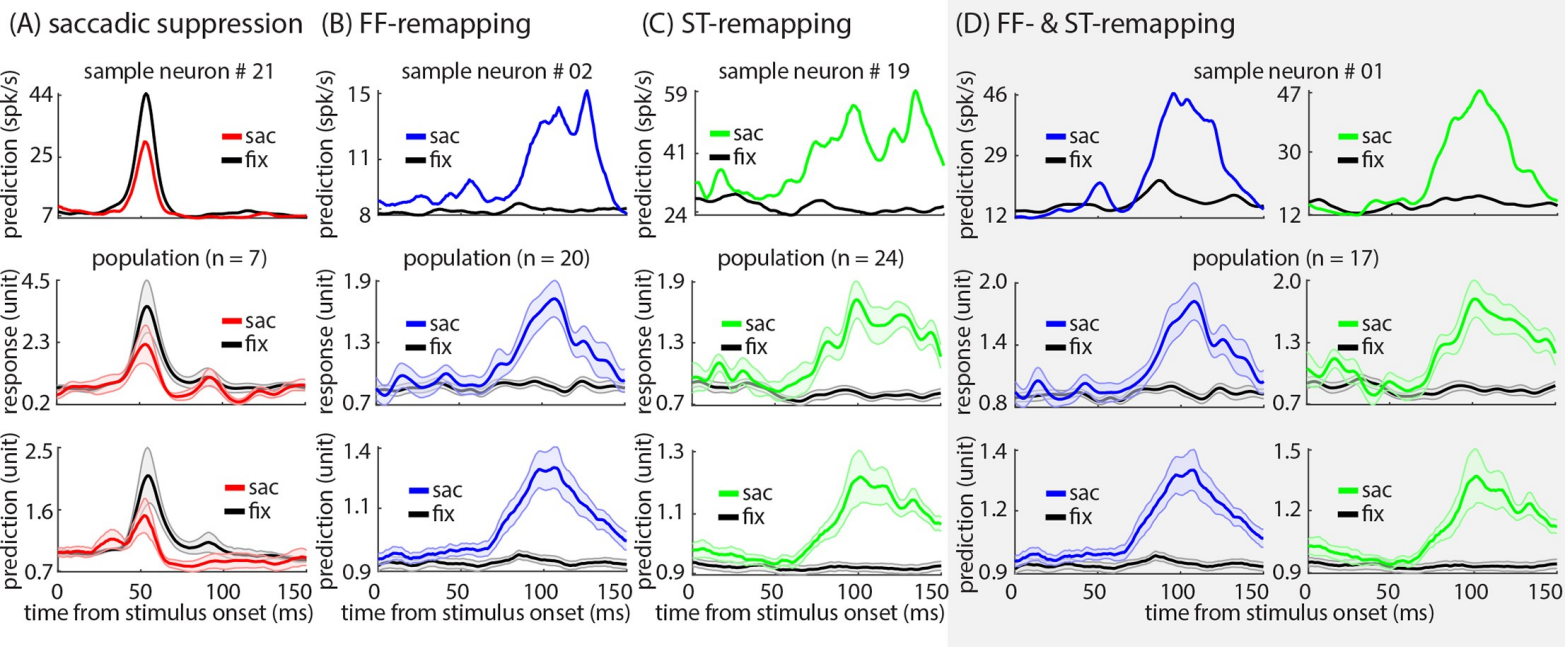
The model reproduces saccadic suppression, FF-remapping, and ST-remapping effects in the individual neurons and the modulated subpopulations. **(A) Model reproduces the saccadic suppression effect**. Top panel shows the model prediction for the same example neuron shown in Fig 1B, for a stimulus presented in its RF during the fixation (black) and perisaccadic (red) periods. Bottom two plots show the average of the actual (middle) and predicted (bottom) responses for 7 cells with significant saccadic suppression in both the response and model prediction. The shaded area indicates the mean ± SE. **(B) Model reproduces the FF-remapping effect**. Top panel shows the model prediction for the same example neuron shown in Fig 1C, for a stimulus presented in its FF during the fixation (black) and perisaccadic (blue) periods. Bottom two plots show the average of the actual (middle) and predicted (bottom) responses for 20 cells with significant FF-remapping in both the response and model prediction. **(C) Model reproduces the ST-remapping effect**. Top panel shows the model prediction for the same example neuron shown in Fig 1D, for a stimulus presented around the ST during the fixation (black) and perisaccadic (green) periods. Bottom two plots show the average of the actual (middle) and predicted (bottom) responses for 24 cells with significant ST-remapping in both the response and model prediction. **(D) Model reproduces the co-occurring FF- and ST-remapping effects**. Top panel shows the model prediction for the same example neuron shown in Fig 1E, for a stimulus presented in its FF and around the ST during the fixation (black) and perisaccadic (blue and green) periods. Bottom two plots show the average of the actual (middle) and predicted (bottom) responses for 17 cells with significant FF- and ST-remapping in both the response and model prediction.

As seen in Fig 6, the model mimics the perisaccadic responses at both the level of individual neurons and the subpopulation of neurons which display each perisaccadic modulation. The model predictions are shown for the same sample neurons depicted in Fig 1B–1E.

Fig 6A, top panel, shows the saccadic suppression effect observed in Fig 1B (left) as predicted by the model. For this neuron, the average model-predicted firing rate over the early response window (50–75 ms after stimulus onset) in response to an RF stimulus dropped from 22.69 ± 0.78 (mean ± SE) spk/s during fixation to 15.72 ± 1.72 (mean ± SE) spk/s when the stimulus appeared just prior to saccade onset (example neuron, p < 0.001). The average of the perisaccadic normalized responses for the subpopulation of neurons with a significant saccadic suppression effect in both the experimental data and model prediction are shown in Fig 6A, middle and bottom panels respectively (n = 7 out of 8). For this subpopulation, the average normalized response over the early response window dropped from 2.48 ± 0.42 to 1.38 ± 0.30 (mean ± SE), and the average predicted firing rate over the same response window decreased from 1.59 ± 0.16 to 1.06 ± 0.09 (mean ± SE). Fig 6B, top panel, shows the FF-remapping effect observed in Fig 1C (left) as predicted by the model. For this neuron, the average model-predicted firing rate over the late response window (80–150 ms after stimulus onset) in response to an FF stimulus increased from 8.01 ± 0.06 (mean ± SE) spk/s during fixation to 11.64 ± 0.36 (mean ± SE) spk/s when the stimulus appeared just prior to saccade onset (example neuron, p < 0.001). The average of the perisaccadic normalized responses for the subpopulation of neurons with a significant FF-remapping effect in both the experimental data and model prediction are shown in Fig 6B, middle and bottom panels respectively (n = 20 out of 23). For this subpopulation, the average normalized response over the late response window increased from 0.89 ± 0.03 to 1.36 ± 0.08 (mean ± SE), and the average normalized predicted firing rate over the same response window increased from 0.93 ± 0.02 to 1.19 ± 0.04 (mean ± SE). Fig 6C, top panel, shows the ST-remapping effect observed in Fig 1D (left) as predicted by the model. For this neuron, the average model-predicted firing rate over the late response window in response to an ST stimulus increased from 24.53 ± 0.39 (mean ± SE) spk/s during fixation to 47.24 ± 2.25 (mean ± SE) spk/s when the stimulus appeared just prior to saccade onset (example neuron, p < 0.001). The average of the perisaccadic normalized responses for the subpopulation of neurons with a significant ST-remapping effect in both the experimental data and model prediction are shown in Fig 6C, middle and bottom panels respectively (n = 24 out of 37). For this subpopulation, the average normalized response over the late response window increased from 0.79 ± 0.03 to 1.48 ± 0.07 (mean ± SE), and the average normalized predicted firing rate over the same response window increased from 0.92 ± 0.02 to 1.19 ± 0.04 (mean ± SE). Finally, Fig 6D, top row, shows the FF- and ST-remapping effects observed in Fig 1E (left column) as predicted by the model. For this neuron, the average model-predicted firing rate over late response window to FF and ST stimuli increased from 17.39 ± 0.38 (mean ± SE) spk/s and 15.85 ± 0.36 (mean ± SE) spk/s during fixation to 32.12 ± 2.50 (mean ± SE) spk/s and 33.11 ± 1.98 (mean ± SE) spk/s when the stimuli appeared just prior to saccade onset respectively (example neuron, p < 0.001 for both). The average of the perisaccadic normalized responses for the subpopulation of neurons with significant FF- and ST-remapping effects in both the response and model prediction are shown in Fig 6D, middle and bottom rows respectively (n = 17 out of 22). For this subpopulation, the average normalized response to FF and ST stimuli over the late response window increased from 0.88 ± 0.03 to 1.43 ± 0.09 (mean ± SE) and from 0.78 ± 0.04 to 1.56 ± 0.09 (mean ± SE) respectively, and the average predicted firing rate in response to FF and ST stimuli over the same response window increased from 0.92 ± 0.02 to 1.22 ± 0.05 (mean ± SE) and from 0.91 ± 0.02 to 1.23 ± 0.06 (mean ± SE) respectively.

**Fig 7:**
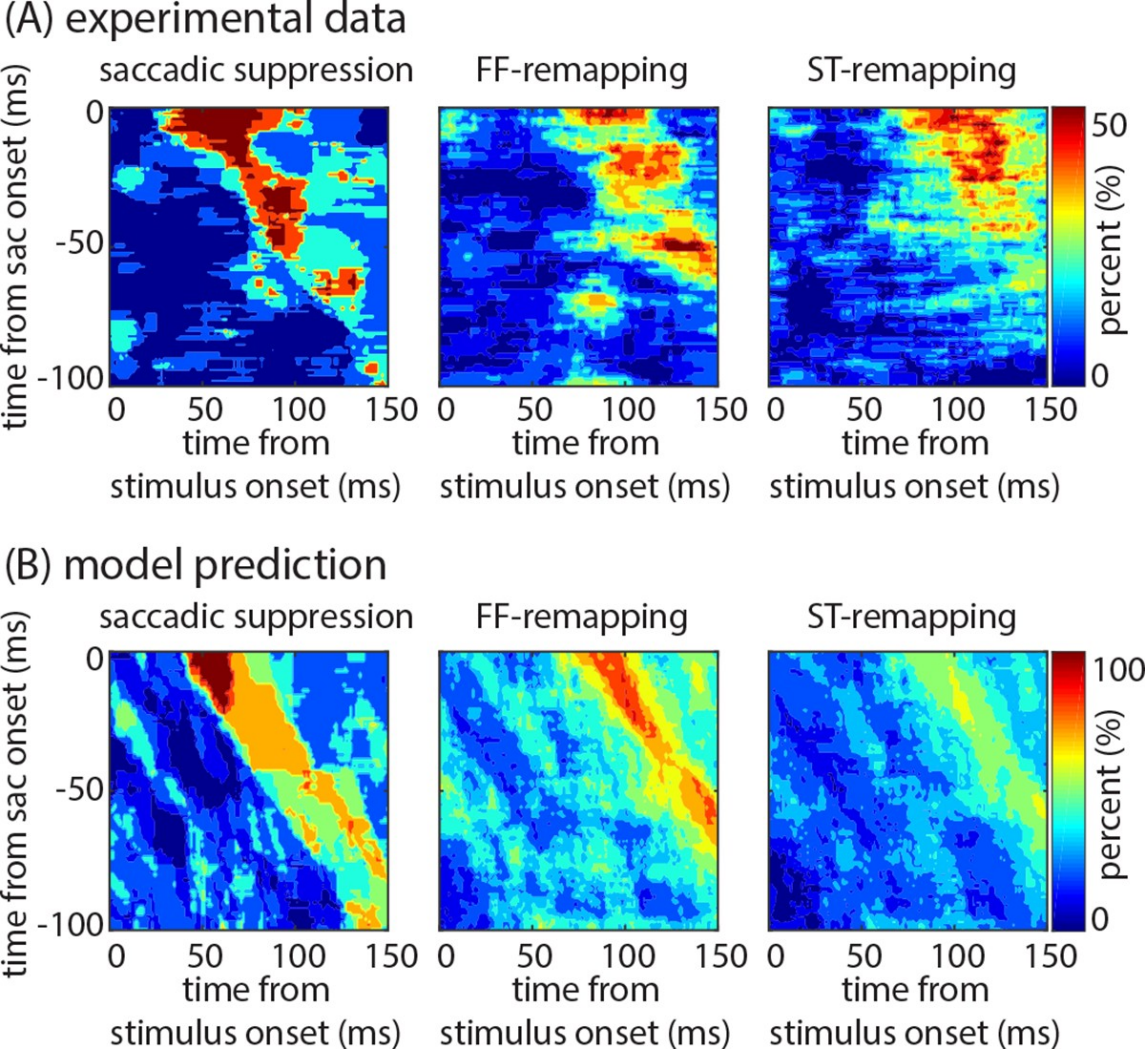
The model captures the time course of perisaccadic effects across the population. **(A) Experimental prevalence of saccadic suppression, FF-remapping, and ST-remapping effects in the neural population, as a function of time**. Plots show the percentage of the population (color) significantly modulated by each factor as a function of time from stimulus onset (x-axis) and time from saccade onset (y-axis). **(B) Model-predicted prevalence of saccadic suppression, FF-remapping, and ST-remapping effects in the neural population, as a function of time**. Plots show the percentage of the model-predicted population response (color) significantly modulated by each factor as a function of time from stimulus onset (x-axis) and time from saccade onset (y-axis).

Next, we compared the actual and predicted prevalence maps of the saccadic suppression, FF-remapping, and ST-remapping effects as a function of time from saccade and from stimulus onset. Fig 7A shows the experimental prevalence maps of saccadic suppression, FF-remapping, and ST-remapping (left to right, respectively); each plot shows the percent of neurons displaying the corresponding effect at each time point relative to saccade and stimulus onset. Fig 7B displays the same prevalence maps based on the model predictions. The frequency of occurrence and the timing of effects across the population are similar for the experimental data and the model prediction. This fact confirms that not only can the model replicate the perisaccadic effects well, but also it follows the dynamics (timing) of those perisaccadic modulations very closely. Strong correlations between the experimental and model-predicted values confirmed the model ability to replicate the timing and frequency of occurrence of effects across the population (saccadic suppression, r = 0.65, p < 0.001; FF-remapping, r = 0.60, p < 0.001; and ST-remapping = 0.62, p < 0.001; Pearson product-moment correlation). As detailed in the Methods section, the experimental prevalence maps are drawn based on the empirical firing rate values obtained by smoothing the observed spike trains, and accordingly have higher variability than the model-predicted firing rate values. This high variability means that fewer effects reach statistical significance, as reflected in the low values of the experimental maps in comparison with the model-predicted ones.

### The model produces predictions about the time course and contribution of perisaccadic modulatory sources to the neuron’s instantaneous firing rate

The model allows us to disassociate the contributions of multiple modulatory sources in generating the instantaneous firing rate of a neuron at each point in time relative to a saccade, which was not possible with any previous computational approach.

**Fig 8:**
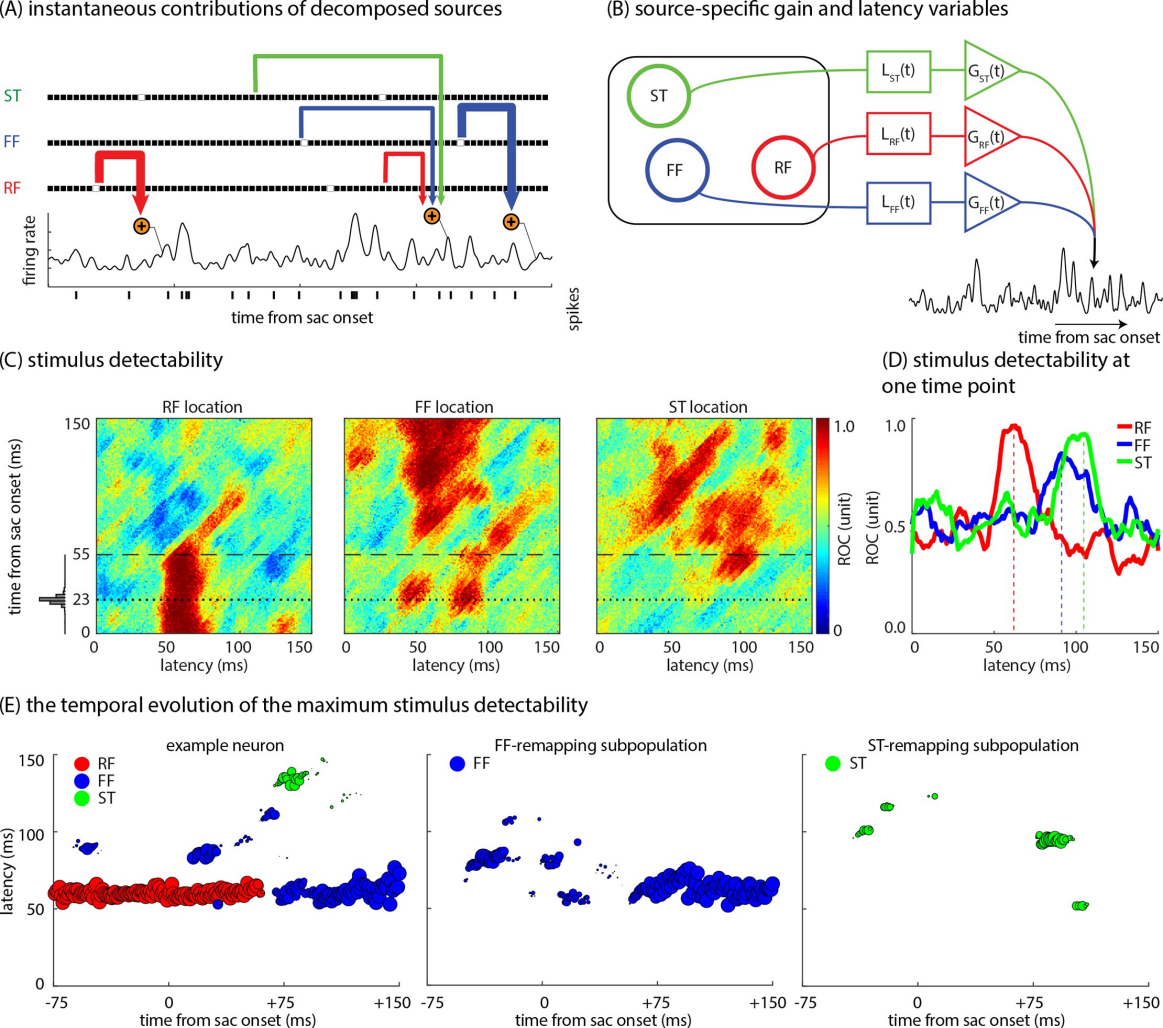
The model separates multiple sources contributing to the neuron’s instantaneous response. **(A) The neural response is due to contribution from stimuli presented at different locations at different time points.** The upper black traces represent the ST, FF, and RF stimuli over time. The bottom black trace represents the instantaneous firing rate of the neuron. Arrows represent the gain and latency with which stimuli presented in ST, FF, and RF contribute to the neuron’s firing rate at specific time points (line thickness represents the gain, and horizontal extent illustrates the latency). The relative widths, and latencies are taken from the model prediction using the spiking data of a sample MT neuron. **(B) Stimuli in the RF, FF, and ST areas each drive the neuron’s response with varying latencies and gains.** This figure illustrates a schematic of how multiple modulation sources contribute to the response generation at each instant of time with time-varying dynamics. The G blocks and L blocks represent respectively the time-varying gain and latency with which each source independently affects the neuron’s instantaneous response. **(C) Spatiotemporal stimulus detectability for an example neuron.** Heatmaps show an example neuron’s ability to detect a stimulus (in terms of area under the ROC curve) over time relative to the saccade (y-axis) and relative to the stimulus (latency, x-axis) for the RF, FF, and ST locations. The dotted line shows the average saccade offset times. The histogram on the vertical axis displays the distribution of saccade offset times. The dashed line represents t = 55 ms, which the cross-section of the heatmaps are plotted in Fig 8D. **(D) Spatiotemporal stimulus detectability for an example neuron at one time point.** Stimulus detectability for the example neuron at different locations, as a function of time between stimulus onset and response (latency), for responses 55 ms after a saccade. Detectability is shown for stimuli appearing in the RF, FF, and ST locations (red, blue, and green respectively). This plot represents a 2D cross-section of the heatmaps on the left (black dashed lines show the cross-section). **(E) The temporal evolution of the maximum stimulus detectability for an example neuron and the population.** These plots illustrate a neuron’s maximum stimulus detectability at different stimulus locations and the latency at which that maximum detectability occurs, over time relative to the saccade. The plot on the left illustrates an example neuron’s ability to detect stimuli at the RF location (red), FF location (blue), and ST location (green) over time. Size of the circles indicates the maximum detectability value reached for that source at a particular time to saccade; y-axis value indicates the latency at which the maximum detectability value occurred. The middle plot illustrates the average magnitude and time course of the FF-remapping effect, for a synthetic neuron based on the statistics of the subpopulation with significant FF-remapping. The plot on the right illustrates the average magnitude and time course of the ST-remapping effect, for a synthetic neuron based on the statistics of the subpopulation with significant ST-remapping.

The contributions of the ST, FF, and RF sources to the neurons’ response are illustrated schematically in Fig 8A and 8B. Fig 8A shows how the three sources contribute differently to the neuron’s firing activity at different times relative to saccade onset. As seen, the evoked response in the neuron at each time point is due to stimuli presented in the ST, FF, and/or RF probe locations at different latencies relative to the response onset. In addition, each source contributes with a specific gain. The latency and gain corresponding to each contributing source vary over time. Fig 8B simplifies this idea in a schematic: multiple modulation sources (here: the ST, FF, and RF sources) contribute to the response generation at each instant of time with time-varying gains and latencies.

Fig 8C illustrates one’s neurons changing ability to detect stimuli presented at different locations over time relative to saccade. To quantify a neuron’s ability to detect stimuli, the receiver operating characteristic (ROC) curve analysis was used: for each location and latency, the detectability of the neuron was defined as the ROC between the model-predicted responses and the trial-shuffled responses (see Methods). The neuron’s maximum detectability at a specific location and the latency at which that maximum occurs correspond, respectively, to the gain and latency values shown in Fig 8A and 8B.

Fig 8C shows the detectability values of an example neuron for visual stimuli appearing in the RF, FF, and ST locations as a function of time relative to saccade and stimulus onset. The neuron can detect stimuli presented in the RF location with a latency of ~60 ms; it loses its ability to detect stimuli in the original RF location after the saccade (~60 ms after saccade onset). At almost the same time that the neuron loses the ability to detect stimuli at the RF location, the neuron becomes able to detect stimuli presented at the FF location with a latency of ~100 ms, and stimuli presented at the ST location with a latency of ~100 ms. Finally, at ~100 ms after a saccade, the neuron can detect stimuli presented in its new RF location (former FF location) with a latency of ~60 ms (the normal latency of MT neurons). In brief, Fig 8C shows that a visual neuron can detect stimuli presented at different locations at different latencies during the perisaccadic period, while it is only sensitive to the stimuli presented at the current RF location at normal latency during the fixation period. In order to make this point clear, Fig 8D examines the stimulus detectability and latency for different locations at a single time point relative to the saccade (responses measured at 55 ms after saccade) for the example neuron. As seen, peak detectability at the FF and ST locations occurs at longer latencies than peak detectability for the RF location; however, peak detectability values for the FF and ST locations are nearly equal to the original RF. So, the stimuli which appeared in the RF ~60 ms earlier and stimuli which appeared in the FF or ST ~100 earlier are equally detectable based on the neuron’s activity at that time point, demonstrating how multiple sources can simultaneously drive the neuron’s response with different latencies and gains.

The temporal evolution of the example neuron’s ability to detect stimuli at different locations, and the latency at which the neural response reflects the presence of a stimulus, are shown in Fig 8E, left panel. In this figure, the maximum detectability and the corresponding latency value for a single neuron for a stimulus presented at the RF, FF, or ST probe location are displayed. The stimulus detectability at the RF, FF, and ST probe locations is represented by the red, blue, and green circles, respectively. The x-axis represents time from saccade onset, and the y-axis represents the latency at which peak detectability occurs. The value of peak detectability at each time point is indicated by the size of circles. As seen, at first, the example neuron can only detect stimuli presented at the RF location ~60 ms earlier (red circles). Next, ~65 ms before the saccade, the neuron can also detect stimuli presented at the FF location ~90 ms earlier (blue circles). This ability to detect FF stimuli disappears 25 ms later, then reemerges ~13 ms after the saccade (with a latency of ~80–100 ms) and persists for ~60 ms. At that point the former FF becomes the RF, and the neuron responds to stimuli in this location with the normal latency of MT neurons, i.e. ~60 ms. Beginning ~80 ms after the saccade, the neuron can no longer detect stimuli presented at the RF location (no more red circles). Meanwhile, from 66 to 130 ms after the saccade, the example neuron detects ST stimuli which were presented ~130 ms earlier (green circles). Taken together, Fig 8E (left panel) confirms that while the neuron can only see the stimuli presented at the (former or new) RF location at normal latency during the fixation period, it can detect stimuli presented at different locations at different latencies during the saccade. To provide a sense of how Fig 8E, left panel, was generated, an animated 3D movie depicting the same information was created (S1 Movie: see Supporting Information). This movie shows the temporal evolution of the maximum detectability and the corresponding latency value for the same neuron shown in Fig 8E, left panel, in response to stimuli presented at the RF, FF, and ST probe locations as a saccade is prepared and executed. The detectability to the RF, FF, and ST locations is represented by the red, blue, and green discs, respectively. In this 3D space, the x and y values represent horizontal and vertical coordinates within the visual field, and the z-axis represents the latency at which peak detectability occurs. Time relative to the saccade changes over time (at a much slower speed), shown by the time label values on top of the plot. The color intensity of the discs represents the peak detectability value.

To understand how the neural detectability of FF and ST stimuli evolves over time in a population of neurons, synthetic neurons were constructed corresponding to the subpopulation average FF- and ST-remapping effects. These synthetic neurons were constructed by averaging the Gaussians of the neurons displaying each effect in both the response and model prediction (as detailed in the Methods section). These synthetic neurons illustrate the population time course of the changes in detectability across the saccade. Fig 8E, middle panel, illustrates how the stimulus detectability at the FF location changes over time relative to a saccade. From ~50 ms before to ~60 ms after a saccade, the population can detect FF stimuli with latencies of ~60–90 ms, indicating that the neural population is detecting stimuli appearing in the FF ~140 to ~0 ms before the saccade. This figure also shows that at around 60 ms after a saccade, the latency of the stimulus detectability at the FF location returns to ~60 ms (i.e., the RF latency value), indicating that the FF has become the new RF. Fig 8E, right panel, illustrates how the population’s ability to detect stimuli presented at the ST location changes over time. Similar to the FF-remapping effect, the population can detect ST stimuli at longer latencies (~100–120 ms) from 45 before to 15 ms after a saccade and from 74 to 114 ms after a saccade. 3D depictions of Fig 8E, middle and right panels, are presented as animated S2 & S3 Movies (see Supporting Information) respectively. These figures (and movies) reveal that FF and ST stimuli contribute to response generation well before saccade initiation, influencing neurons’ responses both during the saccade and after the eyes have landed.

## Discussion

We developed a time-varying model framework capable of precisely capturing fast changes in neuronal responses–induced by dynamic task and behavioral variables–at the resolution of individual neurons, spikes, and trials, as well as dissociating and quantifying the modulatory sources contributing independently to these changes. This new framework yielded several new insights into the coding of time-varying sensory information when applied to the spike trains in the visual cortex measured across rapid eye movements. We tested our model’s ability to capture and dissociate multiple time-varying sources of modulation in the context of perisaccadic changes in visual sensitivity. Neurons’ visual sensitivity changes dramatically around the time of saccades [3–6, 11–16]. The fast timescale on which these changes occur creates challenges both for experimental approaches and for computational models aiming to quantitatively characterize the relationship between various extrinsic or intrinsic system covariates and the modulations in sensitivity to visual input. Our approach combines high spatiotemporal resolution visual stimulation across many locations within the visual field of a neuron with a novel GLM-based model structure in order to accurately capture these fine timescale modulations. The model successfully predicted the responses of visual neurons in area MT, including during the perisaccadic period, at the level of single trials and with a temporal resolution beyond that of existing approaches. Moreover, the model could accurately reproduce the perisaccadic modulations observed in perisaccadic responses, including saccadic suppression, FF-remapping, and ST-remapping, at the level of both single neurons and the population; a goal which, to our knowledge, was unattainable using any previously reported computational framework. These modulations are consistent with previous neurophysiological studies; for example, the FF-remapping effect seen here is consistent with previous reports of memory remapping, but no predictive/anticipatory remapping, in MT [50–52].

The combination of the computational framework designed to capture dynamic changes in sensitivity and a high spatiotemporal resolution stimulus presentation paradigm allowed us to investigate how the spatiotemporal receptive field of a visual neuron evolves over time with a resolution on the order of <10 ms. Importantly, the stimulus presentation paradigm makes no assumptions about the time windows or spatial locations to which neurons will respond, and therefore in comparison with conventional experimental techniques, which mostly rely on a-priori selection of relevant stimulus locations and comparatively large temporal windows, offers both a more complete and higher spatiotemporal resolution picture of dynamic changes in neural sensitivity across saccades. The fitted kernels in the models represent the time-varying spatiotemporal receptive field structure of the neuron as captured by the models. Beyond merely tracking the changes in neurons’ RFs, this research provides a means to quantitatively study how multiple modulatory sources interact in generating the response of a neuron at each moment in time, and accordingly how the visual scene is encoded by neurons. This in turn can provide insight into the problem of how the pre- and post-saccadic scenes are integrated across saccades.

The computational framework developed in this paper opens up a plethora of opportunities for future research and applications; here we discuss some of these possible future uses of the model. First of all, the model can be used to examine the neural basis of psychophysical phenomena observed during eye movements. There are several reports indicating that saccades alter the perception of space and time [53–64]. Several research groups have tried to find explanations for these perceptual changes using abstract models designed to simulate the spiking behavior of visual neurons [21, 65–68]. The present computational framework provides a data-driven, more complete, and quantified description of perisaccadic response modulations, solely based on the statistical features of real spiking data and their relationship with a variety of covariates, allowing examination of the contribution of independent components to various perisaccadic perceptual phenomena. Secondly, the model can provide a read-out of visual information based on neural responses over the time course of a saccade. The choice of pseudorandom visual probe patterns across space and time generated an unbiased estimation of the encoding model, which is crucial for decoding arbitrary temporal and spatial information, which in turn has not been provided to the model during training. This model-based decoding of the spiking activity allows us to directly and quantitatively link neural activity to perception. Thirdly, the decomposition of modulatory effects provides tools for identifying the sources and consequences of their associated modulations. Sources can be selectively eliminated from the model to test their effects on the perceptual read-out, and the effect of source elimination on neural responses can be compared to the results of inactivation experiments. An important question in visual neuroscience is which area or areas in the brain are responsible for the perisaccadic changes in visual neurons. Our model provides an opportunity to explore this question by dissociating sources of modulation, quantifying their strength over time, and correlating them with the activity of various areas of the brain with the aim of finding which area might control each of the modulatory sources, and how inactivation of that area may alter perception across saccades. The current version of the F-model makes some assumptions about the different types of perisaccadic modulatory signals, based on previously reported neurophysiological effects; future work, however, will screen the S-models’ response functions to identify perisaccadic modulations without the assumptions built into the F-model. Finally, the proposed computational framework can serve to generate artificial populations of neurons and test their responses to a larger number of perisaccadic stimuli than would be experimentally feasible, allowing investigation of population-level visual representations. In total, the model presented here provides a blueprint for how sensory stimuli and internal covariates evoke different neural responses due to changes in the neural state. This framework can be applied not only to perisaccadic visual responses, but to a wide variety of brain areas and behavioral contexts in which sensory responses are modulated by combinations of factors such as attentional state, context, reward history, motor preparation, learned associations, and other cognitive variables.

## Methods

### Ethics statement

Two adult male rhesus monkeys (Macaca mulatta) were used in this study. All experimental procedures were in accordance with the National Institutes of Health Guide for the Care and Use of Laboratory Animals and the Society for Neuroscience Guidelines and Policies. The protocols for all experimental, surgical, and behavioral procedures were approved by the Montana State University Institutional Animal Care and Use Committee. Animals were pair-housed when possible and had daily access to enrichment activities. During the recording days, they had controlled access to fluids, but food was available ad libitum. All surgical procedures were carried out under Isoflurane anesthesia and strict aseptic conditions.

### Surgical procedures

Prior to undergoing behavioral training, each animal was implanted with a stainless steel headpost, attached to the skull using orthopedic titanium screws and dental acrylic. All surgical procedures were carried out under Isoflourane anesthesia and strict aseptic conditions. Following behavioral training, custom-made PEEK recording chambers (interior 22 × 22 mm) were mounted on the skull and affixed with dental acrylic. Within the chamber a 22 × 22 mm craniotomy was performed above the extrastriate visual areas including areas V4 and MT (extrastriate craniotomies were centered at −6 mm A/P, 23 mm M/L and −13 mm A/P, 23 mm M/L).

### Experimental paradigm and data acquisition

Monkeys performed a visually guided saccade task during which task-irrelevant random dot stimuli flashed on screen. Two adult male rhesus monkeys were trained to fixate on a fixation point (FP; a red dot) located in the center of the screen. After they fixated, a saccade target (ST; a red dot) appeared 10 degrees away horizontally. Then, after a randomized time interval between 600 and 750 ms (drawn from a uniform distribution), the fixation point disappeared, cuing the monkeys to make a saccade to the ST. After remaining fixated on the ST for 600 ms monkeys received a reward. During this procedure, a series of randomly located probe stimuli were presented on the screen in a 9 by 9 grid of possible locations. Each stimulus was a white square (full contrast), 0.5 by 0.5 degree of visual angle (dva), against a black background. Each stimulus lasted for 7 ms and stimuli were presented consecutively without any overlap, such that at each time point there was exactly one stimulus on the screen. The locations of consecutive probe stimuli followed a pseudorandom order, called a condition. In each condition, a complete sequence of 81 probe stimuli was presented throughout the length of a trial. Conditions were designed to ensure that each probe location occurred at each time in the sequence with equal frequency across trials. The pseudorandom presentation of the probe stimuli made it possible to track the temporal evolution of the neurons’ spatiotemporal receptive field (RF) using an unbiased set of stimuli [69] and independent of their relative timing to the saccade events. For each recording session, the grid of the possible locations of the probes was positioned such that it covered the estimated pre- and post-saccadic receptive fields of the neurons under study, as well as the fixation point and saccade target. The spatial extent of the probe grids varied from 24 to 44.8 (mean ± SD = 30.25 ± 6.13) dva horizontally, and from 16 to 28 (mean ± SD = 18.11 ± 3.97) dva vertically. The (center-to-center) distance between two adjacent probe locations varied from 3 to 5.6 (mean ± SD = 3.78 ± 0.77) dva horizontally, and from 2 to 3.5 (mean ± SD = 2.26 ± 0.50) dva vertically. Each trial lasted between 2100 to 2300 ms. Throughout the entire course of the experiment, the spiking activity of the neurons in area MT was recorded using a 16-channel linear array electrode (V-probe, Plexon Inc., Dallas, TX) at a sampling rate of 32 kHz, and sorted offline using the Plexon offline spike sorter. The spike sorter program was employed to perform a principal component analysis, clusters of spikes with similar waveform properties were manually classified as belonging to a single neuron (single unit). The sorted spikes were then read into Matlab to verify the presence of a visually-sensitive receptive field. From a population of 49 well-isolated neurons, 8 neurons were discarded because they did not respond to any probe stimuli before and/or after the saccade, and the rest were used for analyses. The eye position of the monkeys was monitored with an infrared optical eye tracking system (EyeLink 1000 Plus Eye Tracker, SR Research Ltd., Ottawa, CA) with a resolution of < 0.01 dva, and a sampling frequency of 2 kHz. Stimuli presentation in the experiment was controlled using the MonkeyLogic toolbox [70]. Visual stimuli were presented on a 24-inch ASUS VG248QE LED monitor with a resolution of 1920X1080 pixels with a refresh rate of 144 Hz, positioned 28.5 cm in front of the animal’s eyes. A photodiode (OSRAM Opto Semiconductors, Sunnyvale CA), mounted on the lower left corner of the monitor, was used to record the actual onset and offset times of stimuli appearing on the screen with a continuous signal sampled and stored at 32 kHz. In total, data were recorded from 41 MT neurons during 11 recording sessions. In 9 recording sessions, the saccades were made to the left (27 of 41 neurons), and in 2 recording sessions, the saccades were made to the right (14 of 41 neurons). The positioning of the probe grids, the spatial distribution of the receptive field of the neurons, and the average of the photodiode signal for an example session is provided in the Supporting Information (S1 Fig). The experimental data are available at http://dx.doi.org/10.6080/K0FB514J and https://github.com/nnategh/SFA-Models.

### Receptive field, future field, and saccade target location estimation

The RF location refers to the probe location which generated the maximum firing rate during the fixation period (-500 to -100 ms from saccade onset) in the early response window, i.e. 50 to 75 ms from probe onset. The future field (FF) location was then set to the probe location shifted away from the RF probe in the direction and (rounded) size of the saccade vector. Finally, the saccade target (ST) location was defined as the probe location, out of the 4 × 4 probe locations centered around the ST, which generated the maximum firing rate during the perisaccadic period (-50 to 0 ms from saccade) in the late response window, i.e. 80 to 150 ms after probe onset, compared to the fixation period. To avoid overlap between the ST and FF locations, the FF probe location and all probe locations surrounding it were excluded from the potential ST probe locations (if they fell within the 4 × 4 probe locations centered around the ST).

### Statistical analysis of perisaccadic responses

In this paper, three perisaccadic response modulations were studied: saccadic suppression, FF-remapping, and ST-remapping. Saccadic suppression was defined as a significant decrease in the spike count of the neuron over the early response window (50–75 ms after stimulus onset) in response to a stimulus presented in the RF probe location shortly before a saccade (-30 to 0 ms from saccade onset), compared to the same stimulus presented during fixation (-500 to -100 ms from saccade onset). In the same way, the FF- and ST-remapping in a neuron were defined as increases in the spike count of the neuron in the late response window (80–150 ms after stimulus onset) to a stimulus presented in the FF or ST probe location shortly before a saccade (-50 to 0 ms from saccade onset) compared to the same stimulus presented during fixation (-500 to -100 ms from saccade onset). In all three cases, the statistical significance was tested by comparing the perisaccadic and fixation spike counts (during the same stimulus-aligned window) using the Wilcoxon one-sided signed-rank test, and a p-value of less than 0.05 (with no multiple comparison adjustment) was considered statistically significant.

Note that all statistical analyses were performed on spike counts between windows of the same duration (not estimates of firing rate). For graphical purposes, however, the spike trains were smoothed by convolving with a Gaussian with full width at half-max (FWHM) 13 ms. The response of a neuron to a stimulus presented in a given time interval was estimated by averaging the stimulus-aligned spike trains from 0 to 150 ms after stimulus onset. Due to differences in the mean firing activity of MT neurons, their perisaccadic and fixation responses were normalized by dividing by the grand mean of their firing rates (from 0–150 ms after onset of a stimulus, across both conditions) before averaging over a subpopulation of neurons. The grand mean was defined as the mean of the means of the perisaccadic and fixation responses.

### S-model framework

We developed a sparse-variable generalized linear model framework, termed the S-model, which is able to track the saccade-induced rapid changes in the spatiotemporal sensitivity of the neurons on the order of 7 milliseconds (which is the resolution of stimuli presentation). The principal idea of the S-model is that the stimulus-response relationship in a neuron is characterized by a set of two-dimensional stimulus kernels (*k*(*t*,*τ*)), which represent the spatiotemporal receptive field of the neuron as varying along the time dimension (*t*). Fixing the stimulus kernels along the time dimension results in the conventional one-dimensional stimulus kernels (*k*(*τ*)) used in ordinary generalized linear models (GLMs) [43, 71]. More specifically, the conditional intensity function (CIF) of the S-model, representing the instantaneous firing rate of an MT neuron, i.e., *λ*^(*l*)^(*t*), under our experimental paradigm is described by,

$$\lambda^{\left(l\right)}(t)=f\left(\sum_{x,y,\tau }k_{x,y}(t,\tau ).s_{x,y}^{\left(l\right)}(t-\tau )+\sum_{\tau }h(\tau ).r^{\left(l\right)}(t-\tau )+b(t)+b_{0}\right),$$ (1)

where $s_{x,y}^{\left(l\right)}(t)\in \{0,1\}$ denotes a temporal sequence of probe stimuli presented at probe location (*x*,*y*) on trial *l* where 0 and 1 represent, respectively, an off and on probe condition, *r*^(*l*)^(*t*)∈{0,1} indicates the spiking response of the neuron on that trial, *k*_*x*,*y*_(*t*,*τ*) represents the two-dimensional stimulus kernel corresponding to the stimulus sequence being presented at probe location *(x,y), h(τ)* is the post-spike kernel applied to the spike history, *b*(*t*) is the offset kernel which captures the saccade-induced changes in the baseline activity (activity in the absence of a visual stimulus and feedback from spiking responses) of the neuron over the time course of the experiment, *b*_0_ = f^−1^(*r*_0_) where *r*_0_ is defined as the measured mean firing rate (spikes per second) across all trials in the experimental session, and finally,

$$f(u)=\frac{r_{max}}{1+e^{-u}},$$ (2)

is a static sigmoidal function representing the response nonlinear properties where *r*_*max*_ indicates the maximum firing rate of the neuron obtained empirically from the experimental data. Compared with the empirical nonlinearity estimated nonparametrically from data, this choice of model nonlinearity adequately captured the nonlinear properties of the neurons’ response. All trials were saccade aligned, i.e., *t* = 0 refers to the time when a saccade was initiated. In order to reduce the high dimensionality of the problem, all the kernels were parameterized as a linear combination of a set of basis functions defined across time, delay, or both variables as follows,

$$k_{x,y}(t,\tau )=\sum_{i,j}\kappa_{x,y,i,j}.B_{i,j}(t,\tau ),$$ (3)

$$h(\tau )=-\sum_{i}\eta_{i}^{2}.H_{i}(\tau ),$$ (4)

$$b(t)=\sum_{j}\beta_{j}.O_{j}(t),$$ (5)

where {*κ*_*x*,*y*,*i*,*j*_}, {*η*_*i*_}, and {*β*_*j*_} are the basis parameters of the stimulus kernels, post-spike kernel, and offset kernel respectively. Since the basis functions $\left\{H_{i}(\tau )\right\}$ were set to be positive and the post-spike kernel was set to reflect only the response refractory effects [72], the negative square of the basis parameters {*η*_*i*_} were used such that the resulting *h*(*τ*) produced a non-positive kernel. The basis functions representing the two-dimensional stimulus kernels were set as follows,

$$B_{i,j}(t,\tau )=U_{i}(\tau )V_{j}(t),$$ (6)

where $U_{i}(\tau )$ and $V_{j}(t)$ were chosen to be B-spline functions of order two. $\left\{U_{i}(\tau )\right\}$ span over the delay variable *τ*, representing a 150 ms-long kernel using a set of 26 knots uniformly spaced at {-13, -6, …, 155, 162} ms (in total, 23 basis functions), and $\left\{V_{j}(t)\right\}$ span over the time variable *t*, representing a 1081 ms-long kernel centered at the saccade onset using a set of 159 knots uniformly spaced at {-554, -547, …, 545, 552} ms (in total, 156 basis functions). The basis functions $\left\{H_{i}(\tau )\right\}$ representing the post-spike kernel were chosen to be B-spline functions of order two with non-uniformly distributed 23 knots over the delay variable *τ*; the spacing of the knots around zero, which indicates the spike time, was smaller and increased further away from the spike time and its associated refractory period (the knots were spaced at {1, 2, 3, 4, 6, 8, 15, 22, 29, 36, 43, 50, 57, 64, 71, 78, 92, 106, 120, 134, 148, 162, 176} ms, in total 20 basis functions). Finally, {$O_{j}(t)$} span over the time variable *t*, representing a 1081 ms-long kernel centered at the saccade onset using a set of 77 knots uniformly spaced at {-570, -555, …, 555, 570} ms (in total, 74 basis functions). A visualization of basis functions is presented in the Supporting Information (S2 Fig). From (Eqs 1 and 3–5) together,

$$\lambda^{\left(l\right)}(t)=f\left(\sum_{x,y,\tau ,i,j}\kappa_{x,y,i,j}.B_{i,j}(t,\tau ).s_{x,y}^{\left(l\right)}(t-\tau )-\sum_{\tau ,i}\eta_{i}^{2}.H_{i}(\tau ).r^{\left(l\right)}(t-\tau )+\sum_{j}\beta_{j}.O_{j}(t)+b_{0}\right).$$ (7)

Eq (7) denotes the CIF of the spiking process described by the S-model. The probability of a spike train associated with this Poisson process is thus given by,

$$p(r^{\left(l\right)}|s)=\prod_{t}p(r^{\left(l\right)}(t)|s)\propto \prod_{t}{\left(\lambda^{\left(l\right)}(t).\Delta \right)}^{r^{\left(l\right)}(t)}e^{-\lambda^{\left(l\right)}(t).\Delta },$$ (8)

where ***s*** is the sequence of input stimuli, and ***r***^(*l*)^ = {*r*^(*l*)^(*t*)} represents the sequence of binned spike counts with bins of size Δ ms on trial *l*. Here, the bin size was chosen equal to 1 ms which ensures that at most one spike can fall in each time bin. The point process log-likelihood (*LL*) [44] of the observed spike trains given the model is,

$$LL(\left\{\kappa_{x,y,i,j}\},\{\eta_{i}\},\{\beta_{j}\right\})=\sum_{l,t}\left(r^{\left(l\right)}(t).\log\left(\lambda^{\left(l\right)}(t).\Delta \right)-\lambda^{\left(l\right)}(t).\Delta \right).$$ (9)

{*κ*_*x*,*y*,*i*,*j*_}, {*η*_*i*_}, and {*β*_*j*_} are estimated by maximizing the log-likelihood function given in Eq (9). The choice of nonlinear function in the S-model’s CIF made the problem of log-likelihood maximization non-convex; the block coordinate ascent method was used to solve the optimization problem (detailed later in this subsection).

To avoid overfitting despite the high dimensionality of the S-model, multiple computational approaches were adopted. First, representing each model kernel using a linear combination of smooth basis functions resulted in an optimization process in a lower dimensional space and with well-behaved search paths. Second, a parameter selection strategy was used to identify the subset of parameters most important for mediating the stimulus-response relationship; only those parameters were included in the model fitting procedure, in order to reduce the high dimensionality of the problem. In this strategy, the basis parameters {*κ*_*x*,*y*,*i*,*j*_}, which comprise the majority of the model parameters, were ranked according to their significance in response prediction, and the less significant ones were eliminated by the following procedure: first, the model’s CIF was assumed to be obtained by a single *κ*_*x*,*y*,*i*,*j*_ and with no dependency on the spike train history or the fluctuations in the baseline activity (i.e., without post-spike and offset kernels). To fit this simplified model, an MLE procedure was performed over 100 subsets of the data, each one obtained by randomly selecting 65% of the data (35% as training and 30% as validation) to generate a distribution of the estimated values for *κ*_*x*,*y*,*i*,*j*_. To evaluate the significance of *κ*_*x*,*y*,*i*,*j*_, a control distribution of this parameter was constructed using the same strategy but with a set of shuffled responses. A *κ*_*x*,*y*,*i*,*j*_ parameter was assessed as a significant parameter for response prediction if the mean of its distribution (*μ*_*x*,*y*,*i*,*j*_) satisfied the following condition:

$$|\mu_{x,y,i,j}-{\bar{\mu }}_{x,y,i,j}|\geq 1.5{\bar{\sigma }}_{x,y,i,j},$$ (10)

where ${\bar{\mu }}_{x,y,i,j}$, and ${\bar{\sigma }}_{x,y,i,j}$ are the mean and standard deviation of the control distribution. Those *κ*_*x*,*y*,*i*,*j*_ parameters that were detected as significant were included in the model fitting and all others were set to zero.

Lastly, to help prevent overfitting of the S-model to the training dataset, a cross-validation approach was used to regularize the model parameters. In this approach, the data were randomly split into a training set (35%), a validation set (30%), and a test set (35%). Then, in order to estimate the model parameters, the likelihood function in Eq (9) was maximized using the block coordinate ascent method as follows: (initialization) The model parameters were set to a very small non-zero value (here: 10^−6^, in order to have a non-zero gradient); (step 1) The likelihood function was maximized with respect to the selected parameters (chosen based on the parameter selection strategy detailed above) describing the stimulus kernel corresponding to the probe location (*x*,*y*) = (1,1) while the rest of model parameters were held fixed at their current estimates. The selected parameters were iteratively updated (using only the training data) to maximize the log-likelihood function over both the training and validation data, until the relative change in the root mean of squares of the selected parameters was less than 1%. Then the same procedure was repeated for the parameters describing the stimulus kernel corresponding to the next probe location, until all probe locations were completed. (step 2) the same as step 1, but for the parameters describing the post-spike kernel. (step 3) the same as step 1, but for the parameters describing the offset kernel. (step 4) steps 1–3 were repeated until no update in the model parameters was observed during these steps. Then, the estimation process was terminated, and fitting was considered complete. The test data were withheld from the model fitting procedure and were used to measure the model goodness-of-fit, ensuring the generalizability of the fitted model to unseen data. The block coordinate ascent method gave a stable solution for the data and with regard to different initializations of the parameters, and benefited the convergence time of the estimation procedure.

Employing basis functions as well as the parameter selection strategy reduced the dimensionality of the parameter space; this reduced dimensionality, along with the cross-validation approach, provided a detailed map of the neuron’s time-variant spatiotemporal sensitivity with less concern of overfitting. A visualization of a few sample stimulus kernels, post-spike kernels, and offset kernels obtained from the S-model fitting are provided in the Supporting Information (S3 Fig).

### F-model framework

Although the S-model can capture the dynamics of the spatiotemporal receptive field, and thus can characterize the response modulation on the timescale of a saccade, it does not identify explicitly what sources contribute to the response modulation. The idea of identifying modulatory sources was inspired by the perisaccadic modulations observed in our experimental data and in many previous studies (see Introduction), i.e. saccadic suppression, FF-remapping, and ST-remapping. In fact, the stimulus kernels fitted using the S-model, S-kernels, can be approximated by time- and delay-dependent mixtures of spatial skewed Gaussians where each Gaussian captures the response modulation arising from one of the RF, FF, or ST sources at a given time and delay (for more details, see S4 Fig in the Supporting Information). To quantitatively dissociate the effects of the RF, FF, and ST sources at different times and delays, a factorized sparse-variable generalized linear model, called the F-model, was developed which approximates the fitted S-kernels *k*_*x*,*y*_(*t*,*τ*) by ${\hat{k}}_{x,y}(t,\tau )$ as:

$${\hat{k}}_{x,y}(t,\tau )={\tilde{k}}_{x,y}(\tau )+\sum_{sr}G(x,y;\varphi^{sr}(t,\tau ))+c(t,\tau ),$$ (11)

where ${\tilde{k}}_{x,y}(\tau )$, termed the fixation kernel, represents the average spatiotemporal receptive field of the neuron over the fixation period, defined as

$${\tilde{k}}_{x,y}(\tau )=\frac{1}{t_{2}-t_{1}}\sum_{t=t_{1}}^{t_{2}}k_{x,y}(t,\tau );$$ (12)

where *t*_1_ = −400 ms, and *t*_2_ = −300 ms from saccade; *c*(*t*,*τ*) represents the time- and delay-dependent baseline profile which is uniform across spatial dimensions; and finally, each $G(x,y;\varphi^{sr}(t,\tau ))$ indicates a time- and delay-dependent spatial skewed Gaussian representing the modulation source *sr*∈{*RF*,*FF*,*ST*}, which is parameterized by $\varphi^{sr}(t,\tau )={\{a}^{sr}(t,\tau ),\mu_{x}^{sr}(t,\tau ),\mu_{y}^{sr}(t,\tau ),\sigma_{x}^{sr}(t,\tau ),\sigma_{y}^{sr}(t,\tau ),\rho^{sr}(t,\tau ),\gamma_{x}^{sr}(t,\tau ),\gamma_{y}^{sr}(t,\tau )\}$ as follows,

$$G\left(x,y;\varphi^{sr}(t,\tau )\right)=a^{sr}(t,\tau ).e^{-\frac{1}{2(1-{\rho^{sr}(t,\tau )}^{2})}\{\frac{{\left(x-\mu_{x}^{sr}(t,\tau )\right)}^{2}}{{\sigma_{x}^{sr}(t,\tau )}^{2}}+\frac{{\left(y-\mu_{y}^{sr}(t,\tau )\right)}^{2}}{{\sigma_{y}^{sr}(t,\tau )}^{2}}-2\rho^{sr}(t,\tau ).\frac{\left(x-\mu_{x}^{sr}(t,\tau ))(y-\mu_{y}^{sr}(t,\tau )\right)}{\sigma_{x}^{sr}(t,\tau ).\sigma_{y}^{sr}(t,\tau )}\}}.\Phi \left(\gamma_{x}^{sr}(t,\tau )(x-\mu_{x}^{sr}(t,\tau ))\right).\Phi \left(\gamma_{y}^{sr}(t,\tau )(y-\mu_{y}^{sr}(t,\tau ))\right),$$ (13)

where *a*^*sr*^(*t*,*τ*), $\left(\mu_{x}^{sr}(t,\tau ),\;\mu_{y}^{sr}(t,\tau )\right)$, $\left(\sigma_{x}^{sr}(t,\tau ),\;\sigma_{y}^{sr}(t,\tau )\right)$, *ρ*^*sr*^(*t*,*τ*), and $\left(\gamma_{x}^{sr}(t,\tau ),\gamma_{y}^{sr}(t,\tau )\right)$ represent the amplitude, the *x* – and *y*-coordinate of the center, the horizontal and vertical spread, the orientation, and the horizontal and vertical skewness of the Gaussian kernel $G(x,y;\varphi^{sr}(t,\tau ))$ corresponding to the modulation source *sr* at time *t* and delay *τ*, and Φ(∙)indicates the standard normal cumulative distribution function.

The time- and delay-dependent parameters ***φ***^*sr*^(*t*,*τ*) and *c*(*t*,*τ*) were estimated by minimizing the sum square difference between the F-kernel ${\hat{k}}_{x,y}(t,\tau )$, as specified in Eq (11), and the S-kernel *k*_*x*,*y*_(*t*,*τ*) obtained from the S-model for each time *t* and delay *τ*. In order to eliminate noise included in the S-kernels as well as to alleviate overfitting, the Gaussian parameters were estimated over non-overlapping bins across the delay dimension (instead of single values of delay), i.e. ***φ***^***sr***^ and *c* corresponding to time *t* from saccade and delays *τ*_*b*_≤*τ*<*τ*_*b*+1_ were estimated by minimizing

$$\sum_{x,y}\sum_{\tau_{b}\leq \tau <\tau_{b+1}}{\left({\tilde{k}}_{x,y}(\tau )+\sum_{sr}G(x,y;\varphi^{sr})+c-k_{x,y}(t,\tau )\right)}^{2}$$ (14)

where {*τ*_*b*_} = {1, 20, 40, 50, 53, 56, 59, 62, 65, 68, 71, 74, 77, 80, 85, 90, 95, 100, 105, 110, 115, 120, 125, 130, 135, 140, 145, 151} ms. In order to constrain each Gaussian kernel within an area surrounding the modulation source which the Gaussian kernel represented (i.e. surrounding the RF, FF, or ST probe location as defined before), and to avoid overlap between Gaussian kernels, the Gaussian parameters were subject to a set of bounded limits during the estimation process, such that (1) the center of Gaussian kernels could only move one probe location away from the probe location corresponding to the effect they were intended to represent (i.e., one probe location away from the RF probe location for the center of the Gaussian kernel for the RF, etc.); (2) the horizontal and vertical spread of the Gaussian kernels could not exceed 2 probe locations; (3) their orientations were limited to between -1 and +1; and finally, (4) the absolute values of the horizontal and vertical skewness of the Gaussian kernels were bounded to 5. In order to remove the discontinuities created by estimating the F-kernels over delay bins, the reconstructed F-kernels were smoothed in the delay dimension using a moving average filter with a span of 10 ms. As a result, the F-kernels had lower values in comparison with the corresponding S-kernels, and so, the firing rates predicted by the F-model were lower in magnitude compared to the corresponding S-model.

### Model evaluation

The models’ performance was evaluated over test data, which was used neither for training the model parameters nor for validating the fitted ones, in terms of the log-likelihood of the observed spike trains given the estimated instantaneous firing rate. In order to estimate the instantaneous firing rates, the sequences of stimuli presented to the neuron were given to the model according to Eq (1). To simulate the effect of spiking history, the recorded spike trains were used. Using the true spike history always raises the concern of getting an unfairly good estimate for the model performance due to the presence of a strong refractory period or self-excitatory component in the post-spike kernel. However, neither of these was an issue here: the post-spike kernels were defined to reflect only the refractory effects (as detailed in the “S-model framework” subsection), and in this dataset only a small number of MT neurons fired at rates in which the absolute refractory period came into play. As a result, including the post-spike kernels had no significant impact on the model performance, as demonstrated by comparing the model performance using the true history, the simulated history (as detailed in [73]: algorithm 2), and no history. The results indicated that there is no significant difference in the model performance measures between these three scenarios (S5 Fig in the Supporting Information). Throughout this paper, the true history was used whenever the model was employed to simulate the recorded spiking responses (Figs 3 and 5–7, except Fig 5B).

The log-likelihood evaluates how well the spike times are predicted by the model, and can do so at the level of individual trials. In the log-likelihood formula (see Eq (9)), the first term is larger when the spikes are observed at high values of the estimated firing rate, and the second term is larger when there are fewer spikes at low values of the predicted firing rate. The log-likelihood was normalized by spike counts, as reported in Williamson et al. [74] and Cui et al. [72], to indicate the amount of information being conveyed by individual spikes. One problem with the log-likelihood is understanding the meaning of a given log-likelihood value in isolation. To address this issue, the log-likelihood of the models were compared with the log-likelihood of a NULL model in which the instantaneous firing rate of the neuron was set to its average firing rate; so, the amount of improvement compared to the NULL model normalized by spike counts, i.e. the log-likelihood per spike (Δ*LL*/*spk*), was reported instead of the raw likelihood value.

To prove that the S- and F-models better predict the perisaccadic responses compared to alternative models, the quality of perisaccadic response prediction by the S- and F-models was compared with the N-model -which is the state-of-the-art model in the perisaccadic response modeling as detailed in [48]- in terms of the log-likelihood per spike. For each model, the log-likelihood of the perisaccadic spikes was scatter plotted vs. the log-likelihood of the fixation spikes for the population of 41 MT neurons (Fig 5A). Because the stimuli presented during the perisaccadic period (-50 to 0 ms from saccade onset) evoke responses after 50–150 ms from stimulus onset, the perisaccadic spikes were defined as those spikes observed from 0 to 150 ms after saccade onset. By the same reasoning, the fixation spikes were defined as those spikes observed from 450 to 0 ms before saccade onset.

### A-model framework

In order to robustly capture the fast perisaccadic modulations (saccadic suppression, FF-remapping, and ST-remapping), it is critical to reduce the variability of the model prediction over the limited perisaccadic data; for that purpose, an aggregate version of the F-model, termed the A-model, was developed. The A-model was constructed by (1) fitting the S-model ten times as explained earlier, on different randomly selected subsets of the data, (2) fitting ten F-models corresponding to each fitted S-model, (3) averaging the model variables (***φ***^*sr*^s, and *c*) obtained from each F-model in order to gain a set of aggregate variables, and finally, (4) constructing the A-model kernels using the aggregate variables through Eq (11). Our code for estimating the S-, F-, and A-model parameters and performing model evaluation analysis is provided at https://github.com/nnategh/SFA-Models.

### Statistical analysis of model predictions

After confirming the goodness-of-fit of the S- and F-models over test data, all trials (and not only those withheld for model testing) for each cell were employed for the rest of the analyses in this study to provide more accurate empirical measures from the experimental data. In order to evaluate the models’ ability to capture the perisaccadic effects observed in the experimental data, i.e. saccadic suppression, FF-remapping, and ST-remapping, sequences of stimuli (n = 1000) were presented to the fitted (N-, S-, F-, and A-) models and the corresponding sequences of instantaneous firing rates were generated (based on Eq (1)). The spatial frequency and timing of the stimulus sequences were the same as those for the experimental paradigm. To model the effect of spiking history, the algorithm developed by Chen et al. (algorithm 2, [73]) was employed to simulate the spike history (self-history). Then, the saccadic suppression effect was defined as a significant decrease in the mean firing rate predicted by the model over the early response window (50–75 ms after stimulus onset) in response to a stimulus being presented in the RF probe location shortly before a saccade (-30 to 0 ms from saccade onset) compared to the same stimulus presented during fixation (-500 to -100 ms from saccade onset). The FF- and ST-remapping were defined as significant increases in the mean firing rate predicted by the model in the late response window (80–150 ms after stimulus onset) in response to a stimulus being presented in the FF or ST probe location shortly before a saccade (-50 to 0 ms from saccade onset) compared to the same stimulus presented during fixation (-500 to -100 ms from saccade onset). In all cases, the statistical significance was tested by comparing the perisaccadic and fixation predicted firing rates using the one-sided Wilcoxon rank-sum test. A p-value less than 0.05 was considered statistically significant.

The model-predicted firing rate in response to a stimulus presented in a given time interval was estimated by averaging the stimulus-aligned sequences of firing rates predicted by the model from 0 to 150 ms after stimulus onset. The perisaccadic and fixation firing rates predicted by the model were normalized by dividing by the grand mean of the model-predicted firing rates (from 0–150 ms after onset of a stimulus, across both conditions) before averaging over a population of neurons. The grand mean was defined as the mean of the means of the perisaccadic and fixation firing rates predicted by the model.

### Dichotomous analysis

A dichotomous analysis was performed for each model and for each perisaccadic effect to assess in how many neurons the effect is significantly observed/not observed in both the experimental data and the model prediction (as detailed in the “Statistical analysis of model predictions” subsection). To this purpose, for each model (N-, F-, S-, and A-) and each perisaccadic effect (saccadic suppression, FF-remapping, ST-remapping), a confusion matrix was constructed which reported the presence or absence of the statistical significance of an effect measured from the experimental data versus from the model prediction. The confusion matrix contained the following values: the number of neurons in which the corresponding effect was statistically significant in both the response and the model (true positive, TP), statistically significant in the response but not the model (false negative, FN), statistically significant in the model but not the response (false positive, FP), and statistically significant in neither the response nor in the model (true negative, TN). There are multiple measures to evaluate the overall classification accuracy of a model using a confusion matrix, including sensitivity, accuracy, and precision. The agreement between the experimental data and the model prediction in terms of displaying/not displaying each of the perisaccadic effects was assessed using sensitivity, accuracy, the geometric mean of the sensitivity and precision (GSP), and F-measure (with *α* = 1/2), defined as follows:

$$\mathrm{sensitivity}=\frac{TP}{TP+FN}$$ (15)

$$\mathrm{accuracy}=\frac{TP+TN}{TP+FN+FP+TN}$$ (16)

$$\mathrm{precision}=\frac{TP}{TP+FP}$$ (17)

$$\mathrm{GSP}=\sqrt{\mathrm{sensitivity}.\mathrm{precision}}$$ (18)

$$F‐\mathrm{measure}=\frac{(1+\alpha^{2}).\mathrm{sensitivity}.\mathrm{precision}}{\alpha^{2}.\mathrm{precision}+\mathrm{sensitivity}}$$ (19)

Note that the dichotomous analysis was performed over the entire data set (including the training and validation trials, as mentioned before) in order to use sufficiently large number of trials recorded for each cell for measuring firing rates. The McNemar test [49] was employed to evaluate the statistical significance of different models’ abilities to classify neurons as displaying each of the perisaccadic effects.

### The perisaccadic to fixation performance ratio analysis

In order to evaluate a model’s ability to capture perisaccadic modulations specifically, the perisaccadic performance to fixation performance ratios of the models were calculated and compared. The performance was quantified in terms of Δ*LL*/*spk* (as detailed in the “Model evaluation” subsection), resulting in a unitless performance ratio value. Since the perisaccadic modulations are only measurable if a stimulus appears in one of the RF, FF, or ST probe locations during the perisaccadic period, the perisaccadic and fixation performance values were calculated over those trials from the test data which met this criterion.

The perisaccadic to fixation ratio was employed to compare the S- and F-models. In addition to the S- and F-models, different variants of the F-model were created to assess the contribution of individual sources, represented by time- and delay-dependent spatial skewed Gaussian kernels, to the F-model’s performance. For this purpose, one, two, or all of the sources were eliminated by nulling the parameters (***φ***^*sr*^) of the relevant Gaussian kernel. To null the parameters of a Gaussian kernel, its parameters were replaced by parameters randomly selected from the fixation period (400 to 300 ms before saccade). By eliminating the RF, FF, or ST Gaussian kernel, the F-model without an RF, FF, or ST source was constructed (-RF, -FF, -ST respectively). By preserving only the RF, FF, or ST Gaussian kernel, the F-model with only the RF, FF, or ST source was constructed (+RF, +FF, +ST respectively). By eliminating all of the RF, FF, and ST Gaussian kernels, the F-model with no source was constructed (no-source).

In order to handle outliers created when taking the ratio, the perisaccadic performance values of the population of neurons were regressed as a linear function (with a fixed intercept of zero) of the fixation performance values for a model. The slope of the fitted line (using a robust-fit algorithm to remove outliers) was considered as an estimate for the perisaccadic to fixation ratio for the corresponding model. S6 Fig shows the regression fits and values. For each of the partial models as well as the F-model, the perisaccadic to fixation performance ratio obtained by this way are plotted in Fig 5C, top panel. The error bars indicate the standard errors of the estimated slopes.

Finally, in order to quantify how much adding one source contributes to the performance of the F-model, the increase percentage was defined for the +RF, +FF, and +ST models as the difference between the performance of the corresponding model and the no-source model divided by the difference between the performance of the F-model and the no-source model, stated as a percentage. Also, to quantify how much eliminating one source reduces the performance of the F-model, the decrease percentage was defined for the -RF, -FF, and -ST models as the difference between the performance of the corresponding model and the F-model divided by the difference between the performance of the no-source model and the F-model, stated as a percentage. All instances of the ‘model performance’ here refer to the perisaccadic to fixation performance ratio.

### Prevalence of the perisaccadic effects

The prevalence of the perisaccadic effects at different times relative to a saccade as well as stimulus onset was assessed for both the experimental data and the model prediction (Fig 7).

For the saccadic suppression effect, the prevalence map using the experimental data was constructed as follows: for each neuron, the set of measured firing rate values generated by the neuron in response to a stimulus presented at the RF probe location at time *t* relative to the saccade and latency *τ* from stimulus onset was collected, ${\hat{r}}_{t,\tau }$. In the same manner, the set of measured firing rate values generated by the neuron in response to the same stimulus presented during fixation (-500 to -100 from saccade) was collected for the latency of *τ*, called ${\bar{r}}_{\tau }$. Then, *m*_*t*,*τ*_, a map of 0’s and 1’s, was built for the corresponding neuron:

$$m_{t,\tau }=\left\{ \begin{array}{l} 1,\;if\;{\hat{r}}_{t,\tau }\;is\;significantly\;less\;than\;{\bar{r}}_{\tau }\\ 0,\;otherwise \end{array}\right.$$ (20)

By averaging all *m*_*t*,*τ*_s obtained from those neurons displaying saccadic suppression in both the response and the model prediction, the prevalence map of the saccadic suppression effect was generated. This map illustrates the percentage of the neurons displaying suppression at each time point relative to the saccade as well as to the stimulus onset. The same approach was employed to draw the prevalence map for the FF- and ST-remapping effects using the experimental data, except that *m*_*t*,*τ*_ was built as:

$$m_{t,\tau }=\left\{ \begin{array}{l} 1,\;if\;{\hat{r}}_{t,\tau }\;is\;significantly\;greater\;than\;{\bar{r}}_{\tau }\\ 0,\;otherwise \end{array}\right.$$ (21)

Similarly, the prevalence maps were drawn for each of the perisaccadic effects using the model predictions. In the model-based prevalence maps, the instantaneous firing rate predicted by the model was used instead of the measured firing rate employed in the data-based maps. The instantaneous firing rate of a neuron was measured by smoothing spike trains through convolving with a Gaussian with FWHM 33 ms. The statistical significance was tested by the one-sided Wilcoxon rank-sum test. A p-value less than 0.05 was considered statistically significant. To quantify the similarity between the data-based and model-based prevalence maps for each perisaccadic effect, a Pearson product-moment correlation was used.

### Spatiotemporal detectability analysis

The receiver operating characteristic (ROC) curve analysis [75] was used in this paper to investigate how the spatiotemporal detectability of a neuron changes over time relative to a saccade. For this purpose, sequences of stimuli (n = 1000) were presented to a fitted A-model, and the corresponding sequences of instantaneous firing rates were generated (based on Eq (1)). The spatial frequency and timing of the stimulus sequences were as the same as those for the experimental paradigm. To model the effect of spiking history, the algorithm developed by Chen et al. (algorithm 2, [73]) was employed to simulate the spike history. Then, for each probe location (*x*,*y*), each time point *t* relative to saccade, and each latency *τ*, the set of predicted firing rate values at time point *t* was collected if a stimulus was presented at probe location (*x*,*y*) at time *t*−*τ*; this distribution of firing rates was called **r**_*x*,*y*,*t*,*τ*_. This procedure was repeated after pairing the same sequences of firing rate with shuffled sequences of stimuli, in order to have an estimate of the firing rate distribution when there is no causal relationship between the stimuli and responses (NULL hypothesis); this shuffled distribution of firing rates was called ${\tilde{r}}_{x,y,t,\tau }$. Afterwards, an ROC curve was constructed based on the **r**_*x*,*y*,*t*,*τ*_ and ${\tilde{r}}_{x,y,t,\tau }$ values, and the area under the curve was considered as the detectability of the neuron, *roc*(*x*,*y*,*t*,*τ*), to probe location (*x*,*y*) at time *t* when a stimulus was presented there *τ* ms earlier (or equivalently, with a latency of *τ* ms). The detectability of a neuron indicates how reliably a stimulus presented at a given probe location at a given time and latency can be detected by the neuron. To assess the significance of the detectability value, the ROC between two distinct shuffled firing rate distributions was calculated 100 times to obtain a null detectability distribution. The model-predicted detectability value and null detectability distribution were compared (two-sample t-test), and considered statistically significant if p was less than 10^−9^.

To better visualize the changes in neurons’ detectability over time relative to the saccade, a plot showing the maximum detectability and the corresponding latency over time is shown for an example neuron (see Fig 8E, left panel). For this purpose, at each time point *t* relative to saccade, the maximum detectability of the neuron to each probe location (*x*,*y*), *I*_*x*,*y*,*t*_, and the corresponding latency at which the maximum detectability occurred, *T*_*x*,*y*,*t*_, were determined as follows:

$$I_{x,y,t}=\max_{\tau }\left\{roc(x,y,t,\tau )\right\},$$ (22)

$$T_{x,y,t}={\mathrm{argmax}}_{\tau }\left\{roc(x,y,t,\tau )\right\},$$ (23)

if *I*_*x*,*y*,*t*_ was a statistically significant detectability value. Then, *T*_*x*,*y*,*t*_ for the RF probe location (red), FF probe location (blue), and one of the closest probe locations to the ST (green) were plotted vs. time to saccade, and the maximum detectability reached at each time point, *I*_*x*,*y*,*t*_, was indicated by the size of the markers. *T*_*x*,*y*,*t*_ and *I*_*x*,*y*,*t*_ were smoothed over time with a moving average filter spanning 7 ms. Note that values of *I*_*x*,*y*,*t*_ were included only for corresponding *T*_*x*,*y*,*t*_ values greater than 50 ms, i.e. the maximum detectability had to occur at or after the normal latency of MT neurons. In addition to this plot, an animated movie was created (S1 Movie: see Supporting Information) for the same neuron. In this movie, for each time point *t*, the corresponding *T*_*x*,*y*,*t*_ was visualized by the height of a disc centered at probe location (*x*,*y*) in a 3D space. In this 3D space, the x- and y-axis represent the horizontal and vertical position in the visual field, and the z-axis represents the latency at which the maximum detectability to a probe location occurs. The amount of the maximum detectability, *I*_*x*,*y*,*t*_, is represented by the intensity of the discs’ color. Only the detectability to the three main probe locations displayed in the plot (indicated by color) is shown here.

Finally, in order to have a sense of how the perisaccadic effects were displayed by the population of MT neurons, similar plots and movies were generated for artificially generated neurons representing the population statistics (see Fig 8E, middle and right panels; and S2 and S3 Movies in the Supporting Information). A population analysis was done as follows: the variables (***φ***^*sr*^s, and *c*) obtained from the A-models of the neurons in which a perisaccadic effect was significantly observed in both the response and model prediction were normalized by the distance between the adjacent probes, and averaged. Then, a synthetic neuron was built for each perisaccadic effect using the variables corresponding to the relevant Gaussian kernel (for example, the average of the normalized ***φ***^*FF*^s was used to construct the kernels corresponding to a synthetic neuron representing the FF-remapping effect, while ***φ***^*RF*^s and ***φ***^*ST*^s were set to zeros for that neuron). Then, the same procedure used for the sample neuron was employed to produce a 2D plot as well as an animated movie for that effect (p-value less than 10^−7^ was considered significant). Due to the low number of neurons displaying the saccadic suppression effect in both the response and model prediction (n = 7), this effect was excluded from the analysis.

## Supporting information

S1 Fig Probe grid positions, receptive field locations, and monitor decay. (A) The spatial extent and position of the probe grids (in 11 recording sessions) with respect to the FP (red dot) located at (0,0) dva is displayed here. The position of the probe grids varied from session to session, according to the eccentricity of the receptive field of the neurons recorded in each session, to cover the estimated pre- and post-saccadic receptive fields of the neurons as well as the fixation point and saccade target. (B) The spatial distribution of the receptive fields of the neurons recorded in the sessions where the saccades were made to the left (left panel, n = 27), and to the right (right panel, n = 14) are shown. The receptive fields were mapped by averaging the responses evoked by each probe stimulus presented during fixation period (here, 700 to 140 ms before saccade) over a window 50–70 ms after stimulus onset; the maps of receptive field were interpolated by a factor of 1000, and the contours represent responses at 95% of the maximum level. In the sessions where the saccades were made to the left, the average center of the receptive fields was 5.81 ± 3.63 (mean ± SD) dva rightward, and 5.23 ± 2.08 (mean ± SD) dva downward. In the sessions where the saccades were made to the right, the average center of the receptive fields was 6.49 ± 1.98 (mean ± SD) dva leftward, and 7.44 ± 1.99 (mean ± SD) dva downward. The red arrows represent the saccade vectors. (C) The average of the segments of the photodiode voltage centered around the times at which an ‘off’ command was sent to the monitor is displayed here. As seen, the stimulus offset time is less than 3 ms.
(TIF)

S2 FigVisualization of basis functions.The visual representation of the basis functions used in fitting the S-model, i.e. $\left\{U_{i}(\tau )\},\;\{V_{j}(t)\right\}$, an example $B_{i,j}(t,\tau ),\;\{H_{i}(\tau )\}$, and $\left\{O_{j}(t)\right\}$ (from top to bottom, respectively) are displayed here.(TIF)

S3 Fig Fitted stimulus, post-spike, and offset kernels for three example neurons. The stimulus kernels corresponding to the RF probe location (first row), the stimulus kernels corresponding to the FF probe location (second row), the post-spike kernels (third row), and the offset kernels (fourth row) corresponding to three S-models fitted to the spiking responses of three sample MT neurons (each column) are presented here. The histograms on the vertical axes of the stimulus kernels display the distribution of saccade offset times. The dashed red lines (in offset kernel plots) represent the zero.
(TIF)

S4 Fig S-kernel structure and factorization as a mixture of Gaussians. (A) An S-kernel, *k*_*x*,*y*_(*t*,*τ*), at each time point relative to the saccade onset has a 3D structure (two spatial dimensions, and one delay dimension) which can be approximated by a mixture of several (one to three) spatially skewed Gaussians across the delay dimension, with each Gaussian corresponding to the modulation arising from one of the RF, FF, or ST sources. (B) The F-model captures the spatial sensitivity encoded by the S-kernel at different times and delays. Plots show four 2D cross-sections of a sample S-kernel at different times and averaged over different delay bins (left heatmap in each pair), and the corresponding reconstructions by the F-model (right heatmap in each pair). As detailed in the Methods, the F-kernels were reconstructed over bins across the delay dimension. Panels display the examples of the RF source emerging during the fixation period (top left: t = -300 ms from saccade, τ = 62 to 65 ms), the FF source emerging during the perisaccadic period (top right: t = +80 ms from saccade, τ = 105 to 110 ms), the ST source emerging during the perisaccadic period (bottom left: t = +84 ms from saccade, τ = 130 to 135 ms), and RF and FF sources jointly emerging during the perisaccadic period (bottom right: t = +94 ms from saccade, τ = 74 to 77 ms). The white cross and circle indicate the FP and ST, respectively. The F-model, despite being based on an approximation of the S-model, has a performance of the order of ~60% of the performance of the S-model, in terms of Δ*LL*/*spk* (see Fig 5A).
(TIF)

S5 Fig Comparison of model performance using true, simulated, or no spike history. The model performance using the true history was compared with the model performance using the simulated history, and the model performance using no history. In the model using no history, the spike history term was removed from Eq (1). In the model using the simulated history, the algorithm recently developed by Chen et al. (algorithm 2, [73]) was used to simulate the spike history. Since the F- and A-models shared the same post-spike kernel as the S-model, they were omitted from this analysis, and only those figures demonstrating the goodness-of-fit of the S-model, i.e. Fig 3 and Fig 5A, were regenerated with the models using the simulated history, and using no history, and compared with the model using the true history (i.e., the S-model as used throughout this paper). (A) The predictions of the models using the true history (brown: the same brown traces shown in Fig 3), using the simulated history (cyan), and using no history (magenta) for two representative trials of three sample neurons shown in Fig 3 are compared here. The trials on the top show examples of high prediction accuracy (from left to right, Δ*LL*/*spk* = 1.12, 0.80, and 0.63 bits/spk for the model using the true history; Δ*LL*/*spk* = 1.03, 0.74, and 0.62 bits/spk for the model using no history; and Δ*LL*/*spk* = 1.20, 0.78, and 0.62 bits/spk for the model using the simulated history), and the trials on the bottom show examples of median prediction accuracy (from left to right, Δ*LL*/*spk* = 0.46, 0.41, and 0.19 bits/spk for the model using the true history; Δ*LL*/*spk* = 0.42, 0.35, and 0.20 bits/spk for the model using no history; and Δ*LL*/*spk* = 0.48, 0.39, and 0.20 bits/spk for the model using the simulated history) for each neuron and model. As seen, there is no obvious difference between the models. (B) The scatterplot in the left panel compares the performance of the model using no history vs. the model using the true history, measured in terms of Δ*LL*/*spk*, for 41 neurons during the fixation period. Histogram in the upper right shows the population distribution of change in performance between the model using no history, and the model using the true history during the fixation period. The scatterplot in the middle panel compares the performance of the model using no history vs. the model using the true history, measured in terms of Δ*LL*/*spk*, for 41 neurons during the perisaccadic period. Histogram in the upper right shows the population distribution of change in performance between the model using no history, and the model using the true history during the perisaccadic period. The scatterplot in the right panel compares the performance of the model using no history measured in terms of Δ*LL*/*spk*, for 41 neurons during the fixation and perisaccadic period. Histogram in the upper right shows the population distribution of change in performance between the fixation and perisaccadic period. Arrowheads along the x- and y-axes mark the medians of each distribution. The model using the true history slightly (but not significantly) outperforms the model using no history over the fixation period (left panel: Δ*LL*/*spk* = 0.24 ± 0.02 (mean ± SE) bits/spk for the model used the true history vs. 0.23 ± 0.02 (mean ± SE) bits/spk for the model using no history, p-value = 0.98) and over the perisaccadic period (middle panel: Δ*LL*/*spk* = 0.30 ± 0.02 (mean ± SE) bits/spk for the model using the true history vs. 0.30 ± 0.02 (mean ± SE) bits/spk for the model using no history, p-value = 0.87). The model using no history replicates the result of the model using the true history (Fig 5A, middle panel), and is better at predicting the spike times during the perisaccadic period compared to the fixation period (right panel, p-value <0.001). In all comparisons, the statistical significance was determined by the Wilcoxon signed-rank test (n = 41 neurons). (C) The same as part (B), but for the model using the simulated history. The model using the true history slightly (but not significantly) outperforms the model using the simulated history over the fixation period (left panel: Δ*LL*/*spk* = 0.24 ± 0.02 (mean ± SE) bits/spk for the model using the true history vs. 0.23 ± 0.02 (mean ± SE) bits/spk for the model using the simulated history, p-value = 0.48) and over the perisaccadic period (middle panel: Δ*LL*/*spk* = 0.30 ± 0.02 (mean ± SE) bits/spk for the model using the true history vs. 0.29 ± 0.02 (mean ± SE) bits/spk for the model using simulated history, p-value = 0.99). The model using the simulated history replicates the result of the model using the true history (Fig 5A, middle panel), and is better at predicting the spike times during the perisaccadic period compared to the fixation period (right panel, p-value <0.001). In all comparisons, the statistical significance was determined by the Wilcoxon signed-rank test (n = 41 neurons). There is no significant difference between the results obtained from the true-history model, the simulated-history model, and the no-history model.
(TIF)

S6 Fig Linear regressions for perisaccadic vs. fixation performance. The perisaccadic vs. fixation performance scatterplots for the population of 41 neurons and the corresponding regression lines (dotted lines) are presented here for the partial models as well as the F-model. The slope of the fitted line ± SE, and the p-values indicating the significance of the difference from slope of zero are shown on top of each plot. The dashed-dotted lines represent 1-to-1 line. The regression lines were fitted using a robust linear regression fit using the iterative reweighted least squares method (MATLAB “fitlm” function with robust option using ‘fair’ weighting function) to reduce the effect of outliers.
(TIF)

S1 Movie Evolution of a single neuron's detectability and latency at RF, FF, and ST locations. This movie shows the maximum detectability and the corresponding latency values for a single neuron in response to stimuli presented at the RF, FF, and ST probe locations as a saccade is prepared and executed. The detectability at the RF, FF, and ST locations is represented by the red, blue, and green discs, respectively. The x and y values represent horizontal and vertical coordinates within the visual field, and the z-axis represents the latency at which the peak detectability occurs. The color intensity of the discs represents the peak detectability value. Noticeable events are marked by text descriptions.
(MOV)

S2 Movie FF detectability and latency for a synthetic neuron. This movie shows the maximum detectability and the corresponding latency values for a synthetic neuron -constructed based on the statistics of the subpopulation with significant FF-remapping effect- in response to stimuli presented at the FF probe location as a saccade is prepared and executed. The detectability at the FF location is represented by the blue disc. The x and y values represent horizontal and vertical coordinates within the visual field, and the z-axis represents the latency at which the peak detectability occurs. The color intensity of the disc represents the peak detectability value. Noticeable events are marked by text descriptions.
(MOV)

S3 Movie ST detectability and latency for a synthetic neuron. This movie shows the maximum detectability and the corresponding latency values for a synthetic neuron -constructed based on the statistics of the subpopulation with significant ST-remapping effect- in response to stimuli presented at the ST probe location as a saccade is prepared and executed. The detectability at the ST location is represented by the green disc. The x and y values represent horizontal and vertical coordinates within the visual field, and the z-axis represents the latency at which the peak detectability occurs. The color intensity of the disc represents the peak detectability value. Noticeable events are marked by text descriptions.
(MOV)
