# Supplementary figures and images for: Characterizing and dissociating multiple time-varying modulatory computations influencing neuronal activity
Source: PLoS Comput Biol. 2019 Sep 12;15(9):e1007275. doi: 10.1371/journal.pcbi.1007275 (PMC6759185; doi:10.1371/journal.pcbi.1007275)

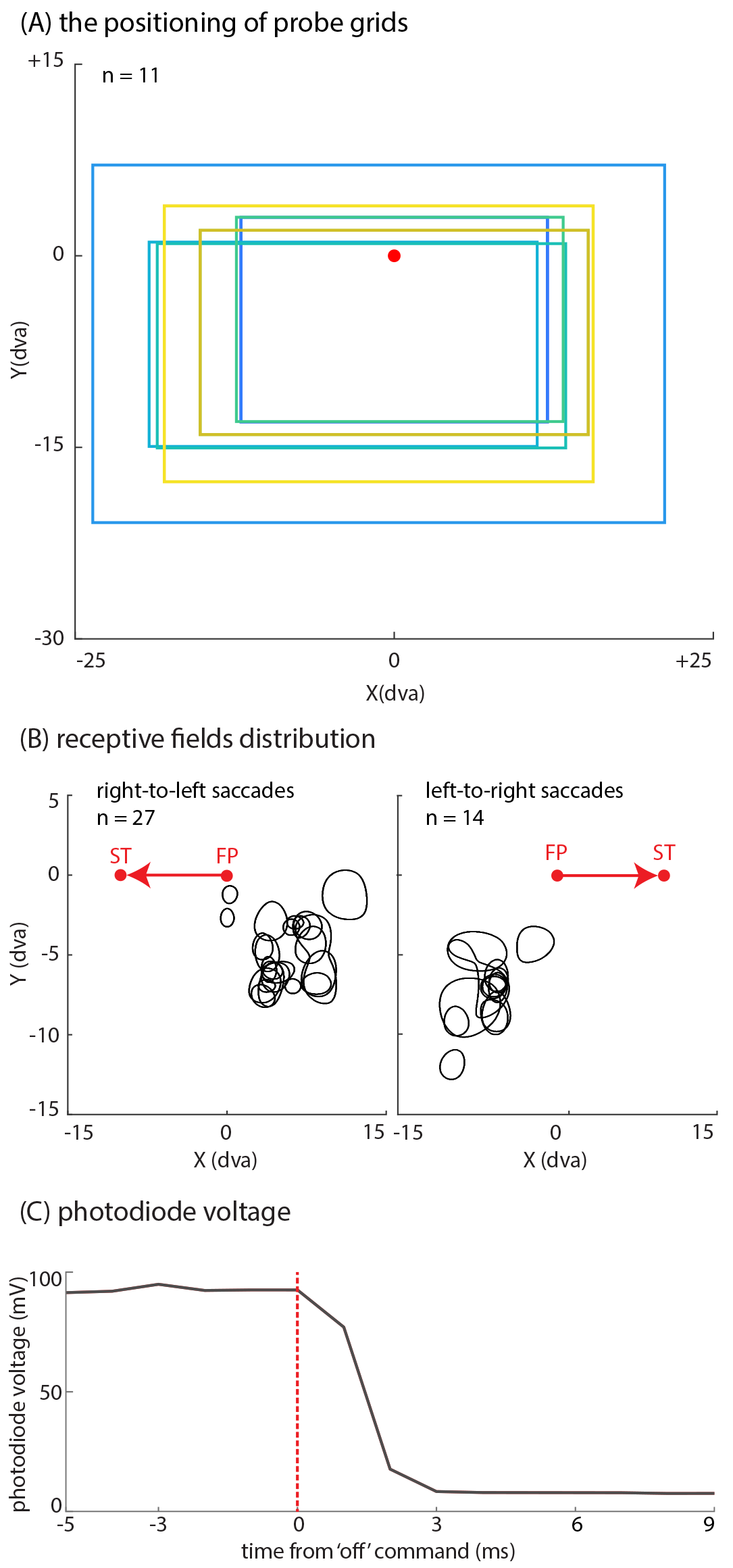

Supplement: S1 Fig — (A) The spatial extent and position of the probe grids (in 11 recording sessions) with respect to the FP (red dot) located at (0,0) dva is displayed here. The position of the probe grids varied from session to session, according to the eccentricity of the receptive field of the neurons recorded in each session, to cover the estimated pre- and post-saccadic receptive fields of the neurons as well as the fixation point and saccade target. (B) The spatial distribution of the receptive fields of the neurons recorded in the sessions where the saccades were made to the left (left panel, n = 27), and to the right (right panel, n = 14) are shown. The receptive fields were mapped by averaging the responses evoked by each probe stimulus presented during fixation period (here, 700 to 140 ms before saccade) over a window 50–70 ms after stimulus onset; the maps of receptive field were interpolated by a factor of 1000, and the contours represent responses at 95% of the maximum level. In the sessions where the saccades were made to the left, the average center of the receptive fields was 5.81 ± 3.63 (mean ± SD) dva rightward, and 5.23 ± 2.08 (mean ± SD) dva downward. In the sessions where the saccades were made to the right, the average center of the receptive fields was 6.49 ± 1.98 (mean ± SD) dva leftward, and 7.44 ± 1.99 (mean ± SD) dva downward. The red arrows represent the saccade vectors. (C) The average of the segments of the photodiode voltage centered around the times at which an ‘off’ command was sent to the monitor is displayed here. As seen, the stimulus offset time is less than 3 ms. (TIF) [file pcbi.1007275.s001.tif]

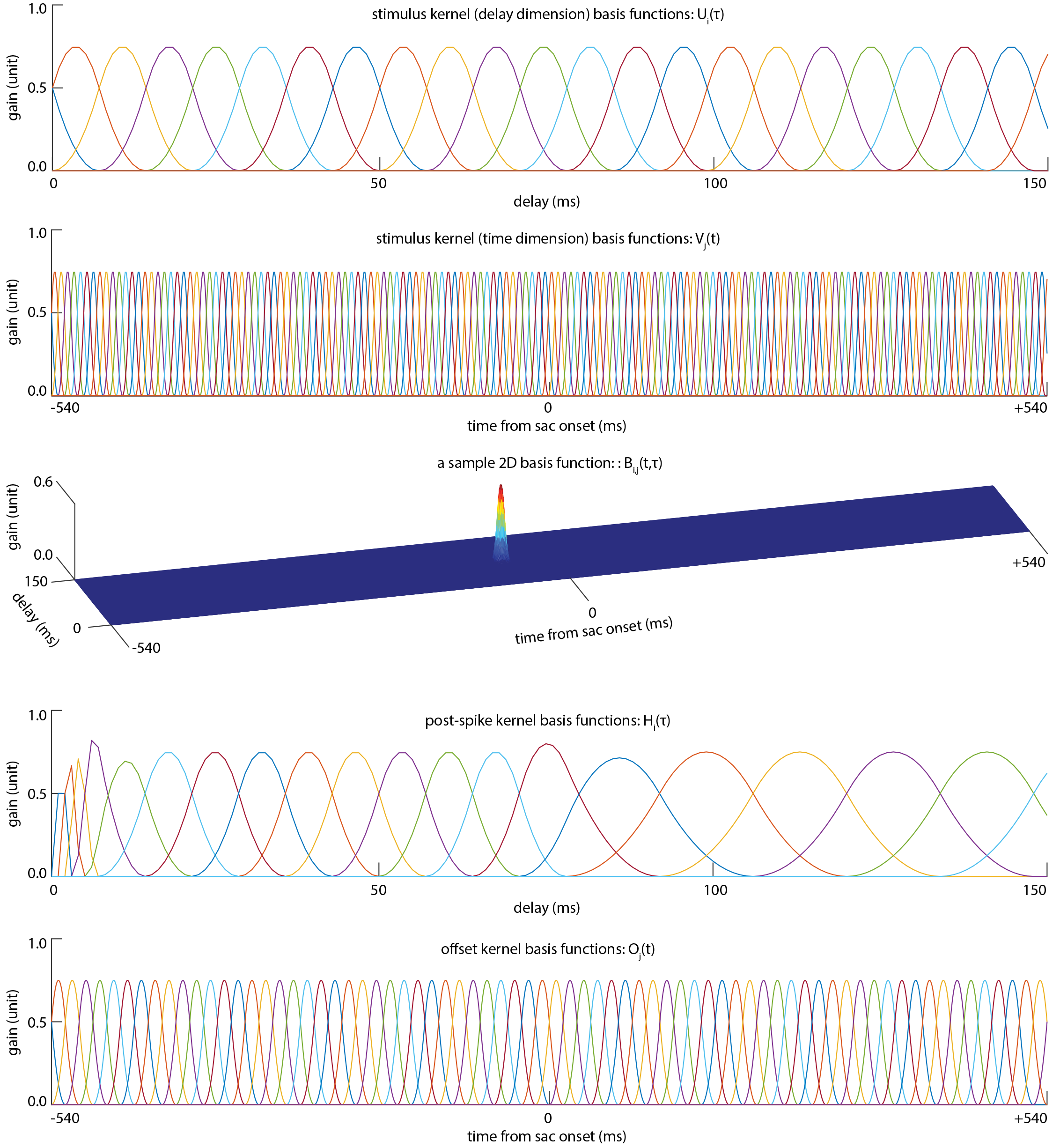

Supplement: S2 Fig — The visual representation of the basis functions used in fitting the S-model, i.e. {Ui(τ)},{Vj(t)}, an example Bi,j(t,τ),{Hi(τ)}, and {Oj(t)} (from top to bottom, respectively) are displayed here. (TIF) [file pcbi.1007275.s002.tif]

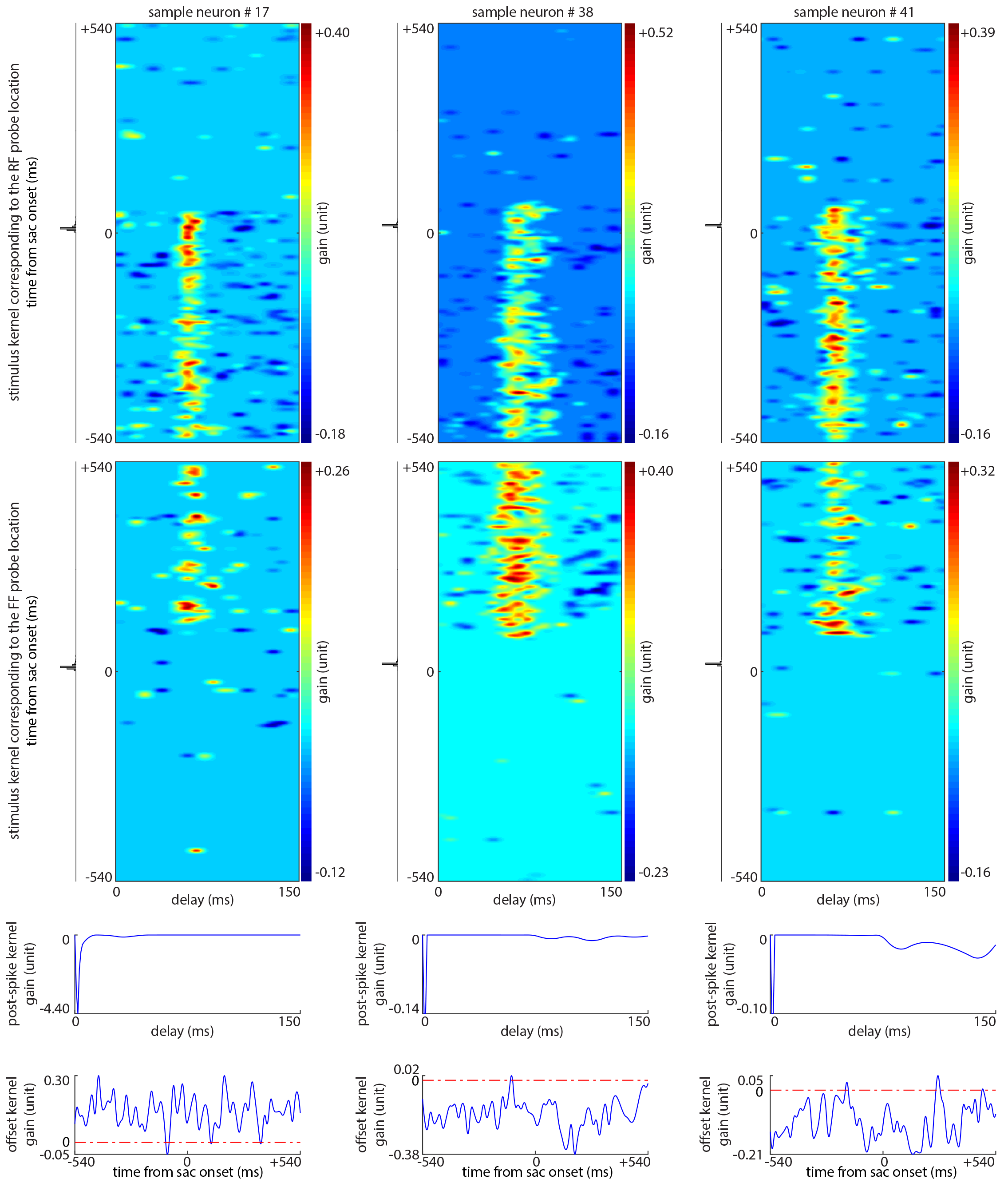

Supplement: S3 Fig — The stimulus kernels corresponding to the RF probe location (first row), the stimulus kernels corresponding to the FF probe location (second row), the post-spike kernels (third row), and the offset kernels (fourth row) corresponding to three S-models fitted to the spiking responses of three sample MT neurons (each column) are presented here. The histograms on the vertical axes of the stimulus kernels display the distribution of saccade offset times. The dashed red lines (in offset kernel plots) represent the zero. (TIF) [file pcbi.1007275.s003.tif]

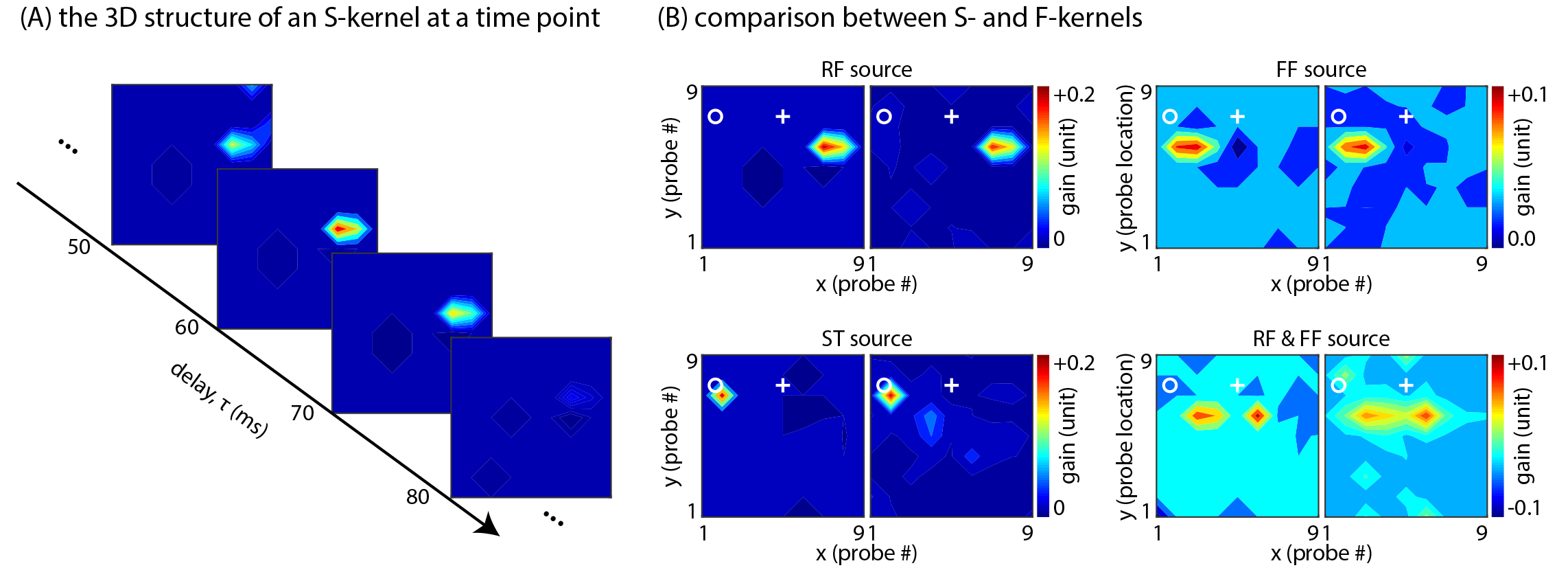

Supplement: S4 Fig — (A) An S-kernel, kx,y(t,τ), at each time point relative to the saccade onset has a 3D structure (two spatial dimensions, and one delay dimension) which can be approximated by a mixture of several (one to three) spatially skewed Gaussians across the delay dimension, with each Gaussian corresponding to the modulation arising from one of the RF, FF, or ST sources. (B) The F-model captures the spatial sensitivity encoded by the S-kernel at different times and delays. Plots show four 2D cross-sections of a sample S-kernel at different times and averaged over different delay bins (left heatmap in each pair), and the corresponding reconstructions by the F-model (right heatmap in each pair). As detailed in the Methods, the F-kernels were reconstructed over bins across the delay dimension. Panels display the examples of the RF source emerging during the fixation period (top left: t = -300 ms from saccade, τ = 62 to 65 ms), the FF source emerging during the perisaccadic period (top right: t = +80 ms from saccade, τ = 105 to 110 ms), the ST source emerging during the perisaccadic period (bottom left: t = +84 ms from saccade, τ = 130 to 135 ms), and RF and FF sources jointly emerging during the perisaccadic period (bottom right: t = +94 ms from saccade, τ = 74 to 77 ms). The white cross and circle indicate the FP and ST, respectively. The F-model, despite being based on an approximation of the S-model, has a performance of the order of ~60% of the performance of the S-model, in terms of ΔLL/spk (see Fig 5A). (TIF) [file pcbi.1007275.s004.tif]

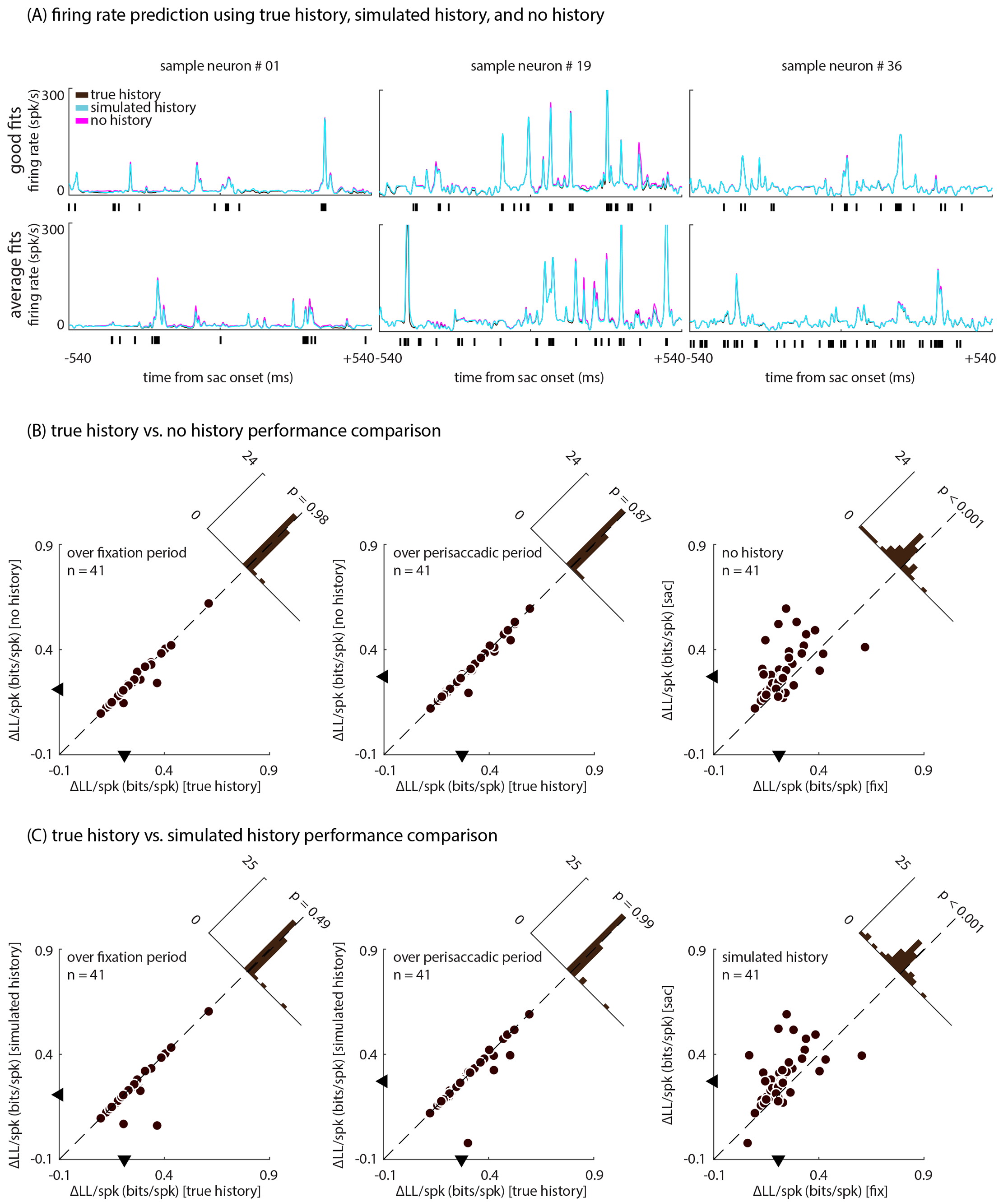

Supplement: S5 Fig — The model performance using the true history was compared with the model performance using the simulated history, and the model performance using no history. In the model using no history, the spike history term was removed from Eq (1). In the model using the simulated history, the algorithm recently developed by Chen et al. (algorithm 2, [73]) was used to simulate the spike history. Since the F- and A-models shared the same post-spike kernel as the S-model, they were omitted from this analysis, and only those figures demonstrating the goodness-of-fit of the S-model, i.e. Fig 3 and Fig 5A, were regenerated with the models using the simulated history, and using no history, and compared with the model using the true history (i.e., the S-model as used throughout this paper). (A) The predictions of the models using the true history (brown: the same brown traces shown in Fig 3), using the simulated history (cyan), and using no history (magenta) for two representative trials of three sample neurons shown in Fig 3 are compared here. The trials on the top show examples of high prediction accuracy (from left to right, ΔLL/spk = 1.12, 0.80, and 0.63 bits/spk for the model using the true history; ΔLL/spk = 1.03, 0.74, and 0.62 bits/spk for the model using no history; and ΔLL/spk = 1.20, 0.78, and 0.62 bits/spk for the model using the simulated history), and the trials on the bottom show examples of median prediction accuracy (from left to right, ΔLL/spk = 0.46, 0.41, and 0.19 bits/spk for the model using the true history; ΔLL/spk = 0.42, 0.35, and 0.20 bits/spk for the model using no history; and ΔLL/spk = 0.48, 0.39, and 0.20 bits/spk for the model using the simulated history) for each neuron and model. As seen, there is no obvious difference between the models. (B) The scatterplot in the left panel compares the performance of the model using no history vs. the model using the true history, measured in terms of ΔLL/spk, for 41 neurons during the fixation period. Histogram in [file pcbi.1007275.s005.tif]

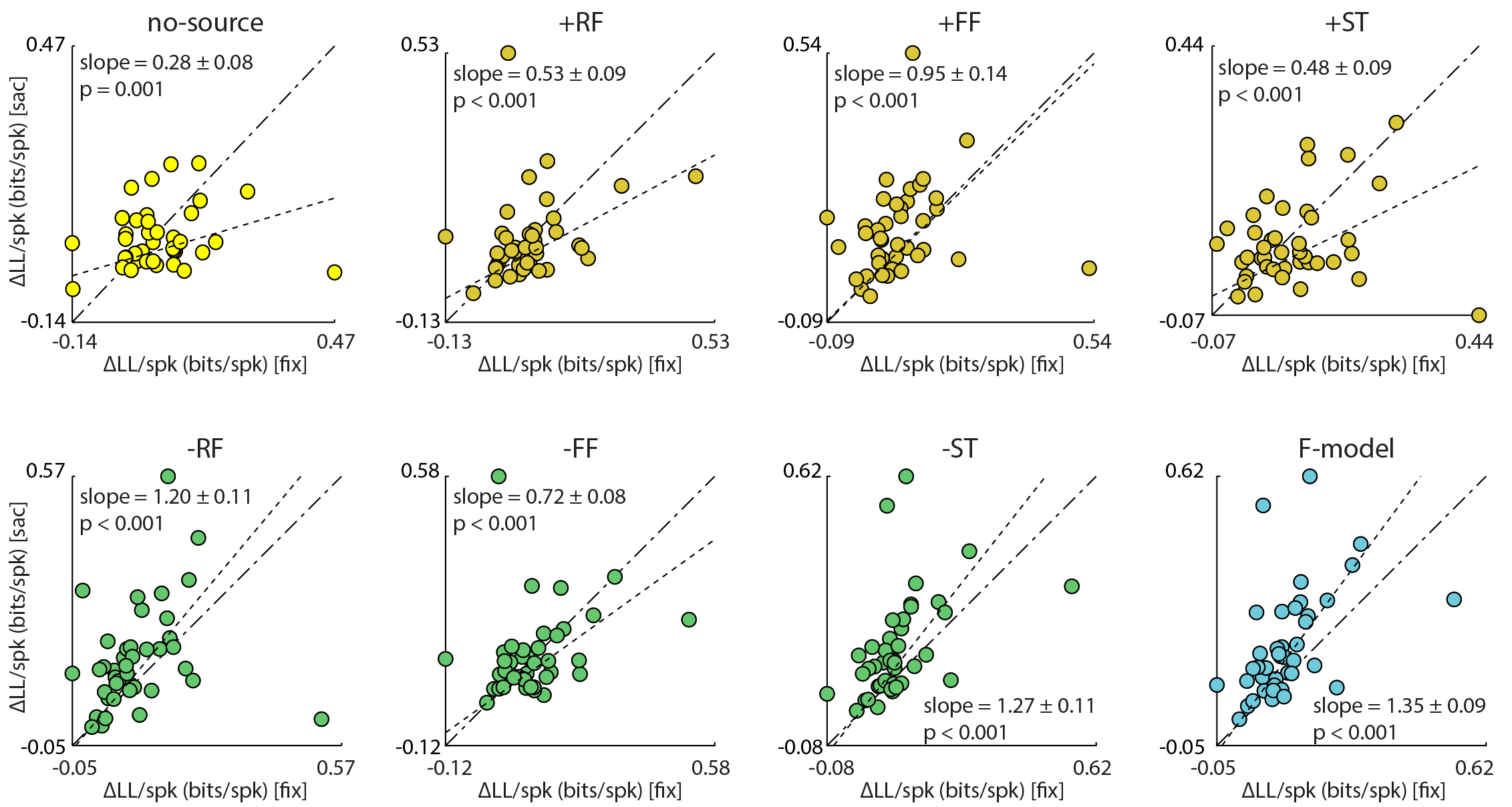

Supplement: S6 Fig — The perisaccadic vs. fixation performance scatterplots for the population of 41 neurons and the corresponding regression lines (dotted lines) are presented here for the partial models as well as the F-model. The slope of the fitted line ± SE, and the p-values indicating the significance of the difference from slope of zero are shown on top of each plot. The dashed-dotted lines represent 1-to-1 line. The regression lines were fitted using a robust linear regression fit using the iterative reweighted least squares method (MATLAB “fitlm” function with robust option using ‘fair’ weighting function) to reduce the effect of outliers. (TIF) [file pcbi.1007275.s006.tif]
